# Thermal activation of Ti(1-x)Au(x) thin films with enhanced hardness and biocompatibility

**DOI:** 10.1016/j.bioactmat.2022.02.027

**Published:** 2022-03-03

**Authors:** Cecil Cherian Lukose, Ioannis Anestopoulos, Theodora Mantso, Leon Bowen, Mihalis I. Panayiotidis, Martin Birkett

**Affiliations:** a Department of Mechanical and Construction Engineering, Northumbria University, Newcastle Upon Tyne, UK; b Department of Cancer Genetics, Therapeutics & Ultrastructural Pathology, The Cyprus Institute of Neurology and Genetics, Nicosia, Cyprus; c The Cyprus School of Molecular Medicine, The Cyprus Institute of Neurology and Genetics, Nicosia, Cyprus; d Department of Applied Sciences, Northumbria University, Newcastle Upon Tyne, UK; e Department of Physics, G.J. Russell Microscopy Facility, Durham University, Durham, UK

**Keywords:** Ti-Au thin film coating, Hardness, Biocompatible, L929 mouse fibroblast, Implants

## Abstract

The lifetime of orthopaedic implants can be extended by coating the softer Ti_6_Al_4_V alloy with harder biocompatible thin films. In this work, thin films of Ti_(1-x)_Au_(x)_ are grown on Ti_6_Al_4_V and glass substrates by magnetron sputtering in the entire x = 0–1 range, before their key biomechanical properties are performance tuned by thermal activation. For the first time, we explore the effect of in-situ substrate heating versus ex-situ post-deposition heat-treatment, on development of mechanical and biocompatibility performance in Ti–Au films. A ∼250% increase in hardness is achieved for Ti–Au films compared to bulk Ti_6_Al_4_V and a ∼40% improvement from 8.8 GPa as-grown to 11.9 and 12.3 GPa with in-situ and ex-situ heat-treatment respectively, is corelated to changes in structural, morphological and chemical properties, providing insights into the origins of super-hardness in the Ti rich regions of these materials. X-ray diffraction reveals that as-grown films are in nanocrystalline states of Ti–Au intermetallic phases and thermal activation leads to emergence of mechanically hard Ti–Au intermetallics, with films prepared by in-situ substrate heating having enhanced crystalline quality. Surface morphology images show clear changes in grain size, shape and surface roughness following thermal activation, while elemental analysis reveals that in-situ substrate heating is better for development of oxide free Ti_3_Au β-phases. All tested Ti–Au films are non-cytotoxic against L929 mouse fibroblast cells, while extremely low leached ion concentrations confirm their biocompatibility. With peak hardness performance tuned to >12 GPa and excellent biocompatibility, Ti–Au films have potential as a future coating technology for load bearing medical implants.

## Highlights

• Combined study on biocompatibility and mechanical performance of Ti–Au films.
• Reports on effect of varying of thermal activation on quality of Ti–Au film structure.
• Clear development of super-hard β-Ti_3_Au phase with in-situ thermal activation.
• Peak hardness value > 12 GPa attained for Ti rich films with ex-situ thermal activation.
• All Ti–Au films highly biocompatible with safe cytotoxic profile against L929 cells.

## Graphical abstract

**Figure:**
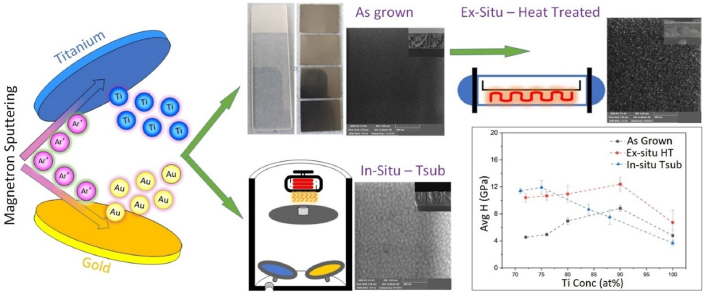

## 1 Introduction

Owing to their excellent biocompatibility, corrosion resistance and mechanical properties, titanium (Ti) and its alloys are used extensively in a variety of orthopaedic implants and fixtures such as: (i) artificial knee and hip joints, (ii) artificial limb systems, (iii) bone plates, (iv) fixature components for fractures, (v) pacemakers and cardiac stents, as well as in (vi) dental support implants (i.e inlays, crowns, overdentures and bridges) [[1], [2], [3], [4], [5], [6], [7]]. The increased need for biocompatible and hard materials is also extended into other systems that interact with the human body in order to provide seamless interaction in the context of comfort and ease, such as articles of jewellery [[8], [9], [10]], artificial prosthesis [11,12] and wearable technology [[13], [14], [15]]. These systems are embedding specific functionality, capable of detecting vital signs including blood pressure, pulse rate, glucose levels and superior mechanical properties among others, ensuring longer term reliability of these advanced systems. In addition, medical and military, organisations are also constantly focusing on developing material systems (i.e. exoskeletons) capable of improving hand-gripping force of patients, and for providing safety and preventing injury of soldiers, when operating under extreme environments [16,17].

However, because of its relative low wear resistance and hardness, there is a great demand for improvement in the mechanical properties and lifetime of Ti and its alloys to make them more suitable for the above-mentioned applications. For example, Ti_6_Al_4_V, which is one of the best biocompatible Ti alloys for fabrication of medical implants [18], suffers from poor tribological performance leading to the formation of fine wear debris when subjected to continuous movement from the surrounding bones [19]. These fine debris containing Al and V can be toxic to the biological system surrounding the implant, further causing its premature loosening [20,21]. These issues can lead to a reduction in implant life and a requirement for early reconstruction surgery. Therefore, it is important that the mechanical hardness of Ti is improved by alloying it with elements which can maintain or even enhance the biocompatibility of Ti itself. Two of the most commonly researched alloying elements to improve the mechanical properties of Ti are silver (Ag) [[22], [23], [24]] and copper (Cu) [22,23,[25], [26], [27]]. Based on their excellent antimicrobial properties both elements can reduce biofilm formation [26] during the initial hours of implant surgery, while they are also known to bring a two-fold increase in mechanical hardness, through the formation of intermetallic phases [[28], [29], [30]]. Ag and Cu ions released by these alloys interact with microbes in the fluid stream to prevent biofilm formation [31]; however, in higher concentrations, these ions can be extremely harmful for the host cells [32,33]. To this end, a search for a safer biocompatible element to alloy with Ti is extremely important.

**Fig. 1:**
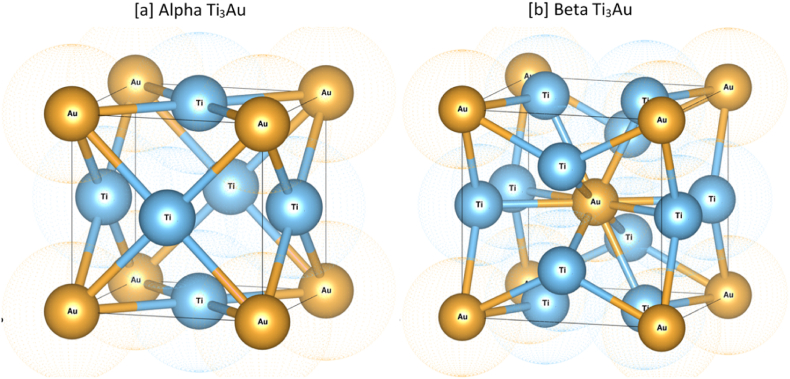
Unit cell structure showing the difference between the coordination configuration of Ti atoms in α and β-phase.

Another highly biocompatible element that belongs to the same family as Ag and Cu is gold (Au) [34]. Recently, a simulative first principle study reported that alloying Au with Ti, can result in an Ti_3_Au intermetallic structure, possessing extremely high hardness [35]. Lee et al. explored the mechanical properties of arc melted Ti–Au alloys in the Au concentration range of 0–40 at % and found hardness to increase from ∼4 to ∼5 GPa with increase in Au doping. However in this study, the presence of a super hard β-phase of Ti_3_Au was not detected [36]. In a similar work by Oh et al., Ti–Au alloys were tested for their cytotoxicity profile and their potential biomedical application in a smaller Au concentration range of 0–21 at %. Specifically, Ti–Au alloys were able to achieve a hardness of ∼3.5 GPa with excellent cytotoxicity results, while a transformation from the α to β-phase with heat treatment of the Ti–Au alloys was also observed [37]. Svanidze et al. also observed the development of a β-phase when arc melting Ti–Au alloys of varying composition were used. Moreover the hardness value of these alloys increased non monotonously with increasing Au concentration, reaching its peak value of 800HV (∼7.85 GPa) around an intermetallic composition, forming cubic Ti_3_Au [28]. Although Au belongs to the same group as Ag and Cu and has the same valency, its higher mass density allows it to exhibit higher valence electron density (VED). When Au is alloyed with Ti, its higher VED results in increased bond strength, providing higher hardness for the resulting alloy [28]. Ti_3_Au starts forming from 10 to 35 at% of Au in the Ti–Au composition ratio and exists in two different forms: α and β. The calculated lattice parameter for the β-phase (∼5.1 Å) is greater compared to that of the α-phase (∼4.1 Å), therefore, the Burgers vector of the unit cell with the larger lattice parameter will exhibit enhanced hardness because of the higher formation energy [35]. Another reason for hardness enhancement is that Au exists in a 12-fold coordination in both α and β-phases, with each Au atom surrounded by 12 Ti atoms, as depicted in Fig. 1. The Ti atoms in the α-phase are also in a 12-fold coordination, as each Ti atom is surrounded by 8 Ti and 4 Au atoms (Fig. 1a). However, in the β-phase (Fig. 1b), the Ti atoms exist in a 14-fold coordination system with 10 Ti atoms and 4 Au atoms surrounding them, resulting in better packing of the unit cell. This difference between the coordination of Ti and Au atoms within the tightly packed unit cell, presents a higher energy barrier for dislocation slipping within the lattice system, resulting in higher hardness for the Ti_3_Au β-phase compared to the α-phase [28]. However, all the previously discussed studies were carried out on bulkTi_3_Au samples created by arc melting of Ti and Au in weighed proportions [38].

Karimi et al., proposes that deposition of the Ti_3_Au alloy in thin film format will be advantageous to overcome the embrittlement issue arising because of the Pugh criterion (B/G < 1.75) [9,35,39]. While β-Ti_3_Au possesses improved hardness and lower coefficient of friction, its limited number of easy slip systems, larger Burgess vector, restricted cross slips and finally, its inability to transfer slips across grain boundaries, weakens the ductility of this material system and makes it brittle [9,35]. von Mises yield criterion requires a minimum of five slip systems for a grain to be subject to deformation and general yielding, whereas Ti_3_Au belongs to the A15 type lattice structure with reported slip activities along three independent slip systems. Therefore, thin film deposition of the Ti_3_Au system as a coating over an underlying substrate like Ti_6_Al_4_V, will reinforce the integrity of the film. Moreover, depositing the Ti_3_Au system as a thin film coating over a Ti_6_Al_4_V implant will significantly reduce the amount of expensive Au material required when compared to manufacturing a complete implant from Ti_3_Au alloy.

The selective coating of implants is also beneficial to overcome the stress shielding effect, which is a key reason for early failure of implants [40,41]. Stress shielding is a phenomenon whereby the implant will bear the weight of the body due to a mismatch of stiffness between natural bone (∼30 GPa) and common implant materials (110–230 GPa), leading to bone resorption and aseptic loosening of the implant [41]. Stress shielding is strongly influenced by the choice of material system employed at areas of the implant that are in contact with the bone, like proximal, mid and tip areas of an artificial hip implant [41] and coating these regions of a Ti_6_Al_4_V alloy implant with Hydroxyapatite, a calcium phosphate based mineral with chemical similarity to bone [[42], [43], [44]] is proven to be effective at reducing this phenomena. On the other hand, hard wearing articulating surfaces that are not in contact with bone, such as the femoral head and socket regions of a Ti_6_Al_4_V hip implant, could be coated with a super hard Ti–Au based alloy to extend its lifetime and reduce the requirement for expensive reconstruction surgery [45]. This selective area coating with Ti–Au would also further reduce the amount of expensive Au material required on the implant.

Karimi et al. also observed that development of intermetallic phases in the Ti–Au system is heavily dependent on thermal energy. The alloy systems undergo structural changes when heat treated at elevated temperature and the β-phase of Ti_3_Au was reported to emerge at temperatures higher than 400 °C. Precipitation of finely dispersed β-phase from the supersaturated α-phase can occur at high temperature leading to formation of pinning sites, which will lead to precipitation hardening of the as-grown α-phase of Ti_3_Au thin films [36]. Thermal energy can be introduced to a thin film either in-situ while the film is growing in the deposition chamber, or ex-situ via post-deposition heat treatment. For the in-situ method, a heater is placed directly behind the substrate to heat it to the required set point during the deposition process and is an important variable in the growth of thin films, as shown by Thornton's structure zone model [46]. On the other hand, the ex-situ method involves heat treating the thin films after deposition with an external furnace set at the required temperature. The furnace environment can be open air, a vacuum or filled with inert or selective reactive gases and these parameters can have a significant effect on the resulting chemical composition and mechanical performance of the thin films [[47], [48], [49]].

Ti–Au Previous studies on the Ti–Au material system have investigated its structural, mechanical and electronic properties in bulk format and very few publications have reported on the mechanical hardness of this system in thin film format. However, so far, no attempt has been made to investigate the combined biocompatibility and mechanical hardness of this alloy in the entire Ti–Au composition range in thin film format and the potential to improve these properties via thermal activation. Therefore, to the best of our knowledge, this paper presents the first work carried out to investigate the combined mechanical as well as biomedical properties of as-grown thin films of Ti–Au alloy in the composition range extending from pure Ti to pure Au in incremental steps of 15 at.% of Au using ex-situ heat treatment at an elevated temperature of 450 °C. This work also studies the effect of in-situ substrate heating on the quality of Ti–Au thin films and compares the results to films heat treated ex-situ.

## 2 Materials and methods

### 2.1 Thin film deposition

**Fig. 2:**
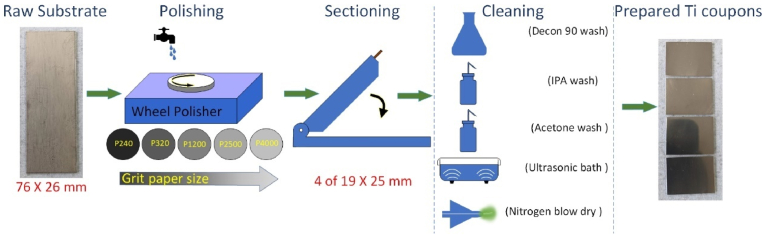
Preparation stages for Ti_6_Al_4_V substrates prior to sputter deposition in NanoPVD chamber.

The Ti_(1-X)_Au_(X)_ (X = 0–1) thin films were prepared by co-sputtering 2-inch diameter targets of 99.99% pure Ti and Au (supplied by Pi-Kem ltd. UK) using a nanoPVD sputter deposition system from Moorefield Nanotechnology. Laboratory standard soda lime glass slides (76 mm by 26 mm) together with Ti_6_Al_4_V of the same size were used as substrates, see Fig. 2. The Ti_6_Al_4_V substrates were polished using SiC abrasive papers from P240 to P4000 to achieve an average surface roughness value better than 40 nm measured in either direction with a line width of 3 mm using an Alicona Infinity Focus surface measurement system. The substrates were then cut into three equal size test coupons of 25 by 19 mm and then thoroughly scrubbed with 1:5 Decon 90 solution and ultrasonically cleaned in DI water, followed by rinsing in IPA, acetone and DI water respectively. The wet coupons were then blow dried with flowing N_2_ before being loaded into the vacuum chamber onto substrate plates with a target to substrate distance of 100 mm and rotated at 5 rpm without any substrate heating. A strip of Kapton tape was applied on a glass substrate to create an artificial step, to be used for profilometer measurement of film thickness post deposition. The chamber was pumped to a base pressure better than 5 × 10^−4^ Pa and 10 sccm of Argon (Ar) was introduced to achieve a working pressure of 0.6 Pa. The Ti target was connected to an DC power source and the Au target was connected to a RF power source, in order to slow down the sputtering rate of Au which is 4–5 times faster than Ti. By varying the power on the RF and DC power sources, ten Ti_(1-X)_Au_(X)_ thin film samples with chemical compositions varying in the range from x = 0 to 1 were achieved. The as-grown samples were then ex-situ heat treated in a Carbolite Gero three zone tube furnace with a tube diameter of 45 mm, sealed at both ends, with flowing Ar gas introduced at a rate of 1 litre/min. Each zone was maintained at 450 °C while samples were within the tube and allowed to cool overnight to room temperature before their removal. After characterization of the as-grown and ex-situ heat-treated samples, selected Ti–Au compositions from the Ti rich zone were deposited with elevated in-situ substrate heating of 450 °C inside the chamber, using a calibrated substrate heater. Thus, in total three series of Ti–Au thin film samples were produced: (1) ‘as-grown’ - thin films grown at room temperature with no additional thermal processing; (2) ‘ex-situ HT’ - thin films grown at room temperature and then heat treated in a tube furnace at 450 °C in flowing Ar; (3) ‘In-situ Tsub’ - thin films grown at elevated substrate temperature of 450 °C inside the deposition chamber with no additional thermal processing.

### 2.2 Thin film characterization

SEM images for surface morphology characterization were collected using a Tescan MIRA 3 system operated at 5–10 keV and compositional information was collected using the built in Oxford instruments X-Max 150 energy dispersive X-ray spectroscopy detector. Film Thickness was measured from SEM cross-sectional images and verified by comparing with the average value of film thickness measured across 5 points of the artificially created film step on the glass substrate, using a Dekatak stylus profilometer. For cross section preparation of samples, focused ion beam (FIB) milling was performed using a FEI Helios Nano Lab 600 Dual Beam system, equipped with a focused 30 keV Ga liquid metal ion source and then imaging of these cross sections were carried out using Secondary Electron, In-Lens detector at 5Kev and 0.34 nA. A Rigaku Smart Lab 2 X-ray diffractometer with Cu K_α_ radiation in parallel beam configuration, was used to collect X-ray reflections between 2θ values of 10 and 80°. Quantitative phase analysis of diffraction patterns was performed by modelling them in a whole powder pattern fitting (WPPF) numerical program based upon the Rietveld refinement method. Nanoindentation was performed using a Hysitron TI900 system with a 3-sided Berkovich tip. Hardness and elastic modulus were measured using the last unloading leg of force displacement curves achieved from the Oliver-Pharr indentation method. For each sample, a 4 × 4 indentation pattern with a spacing interval of 10 μm was made, with maximum indentation depth reaching around 10% of film thickness. The surface roughness of the deposited films was measured using a Nanoveeco dimension 3000 AFM in contact mode using a silicon tip of 15 nm on a surface area of 3 by 3 μm.

### 2.3 Cell lines and reagents

L929 cells (murine fibroblasts) were purchased from Deutsche Sammlung von Microorganismen und Zellkulturen (DSMZ – Braunschweig, Germany). Cells were cultured in Dulbeccos's Modified Eagle Medium (DMEM), high glucose, supplemented with 10% fetal bovine serum (FBS), 2 mM l-glutamine, 100U/ml penicillin and 100 μg/ml streptomycin, while incubated under humidified conditions at 37 °C and 5% CO2. L929 cells were grown as monolayer cultures, while when confluency reached 80–90%, cells were sub-cultured for a maximum of 20–25 passages, before new vials were used. Cell culture media and reagents [FBS, antiobiotics, trypsin, Phosphate Buffer Saline (PBS)] were purchased from Biosera (Kansas City, MO, USA). Resazurin sodium salt was obtained from Fluorochem (Derbyshire, UK). while cell culture plastic ware was obtained from Corning (NY, USA).

### 2.4 Ti–Au thin film and control coupon extracts preparation

Two experimental protocols were applied to obtain extracts from the Ti–Au thin films and polished Ti_6_Al_4_V and Cu coupons of the same dimensions, which were used as positive and negative experimental controls, respectively. Initially, extracts from all three types of Ti–Au thin films (as-grown, ex-situ HT and In-situ Tsub) and Ti_6_Al_4_V and Cu control coupons, were prepared by immersing the test samples into 6-well plates containing 6 ml of DMEM solution for 72 h in a humidified incubator at 37 °C, 5% CO_2_. A light agitation was applied to the 6-well plated containing the extracts for 5 s at the beginning and the end of the preparation period. In another set of experiments, following completion of 72 h immersion of all the test samples in 6-well plates, the ex-situ HT and In-situ Tsub Ti–Au thin film samples and Ti_6_Al_4_V and Cu control coupons were further incubated for an additional 96 h (total 168 h), before the leached culture media was used for cytotoxicity tests on L929 cells. the ex-situ HT and In-situ Tsub samples were tested for a prolonged duration to establish the if thermal crystallization of the Ti–Au thin films will cause an increase in ion leaching thereby adversely affecting cell viability.

### 2.5 Cytotoxicity and biocompatibility characterization of Ti–Au thin films on L929 mouse fibroblast cells

The biocompatibility study was performed in accordance with the standards of ISO 10993 using the Alamar Blue assay to assess the cytotoxicity of the Ti–Au films on L929 mouse fibroblast cells [[50], [51], [52]]. Briefly L929 mouse fibroblasts at a density of 2000 cells/well were seeded in 100 μl/well into 96-well plates and incubated overnight. The next day DMEM cell culture media was replaced with culture media containing Ti–Au thin film leached extracts obtained from either 72 h or 168 h of extract preparation as previously described. For negative control conditions, cells were exposed to complete DMEM media (BLANK), as well as leached media from the Ti_6_Al_4_V coupons, while positive control samples consisted of cells incubated with leached culture media from the Cu coupons and 10% DMSO. In all cases L929 cells were exposed to leached media from the Ti–Au thin films and positive and negative controls for a total of 72 h. At the end of the incubation period, 10 μl of resazurin (1 mg/ml final concentration) was added in each well and the cells were incubated for 4 h at 37 °C. Finally, absorbance measurements were performed at 570 nm and 590 nm (reference wavelength) using an absorbance plate reader (Labtech LT4500, UK). Cell viability levels were then calculated and expressed as percentage of untreated (BLANK) cells.

The remaining quantity from the Ti–Au films and Ti_6_Al_4_V and Cu control coupon extracts were tested in a Perkin Elemer Optima 8000 inductively coupled plasma optical emission mass spectrometer (ICP-OEMS), to measure the level of ions leached in the extracts. Standards were prepared for the range of 1–10 ppm to identify dissolved Ti, Al, V, Cu and Au extracted from Ti–Au thin films or from the Ti _6_Al_4_V and Cu control coupons.

## 3 Results

### 3.1 As-grown vs ex-situ heat treated Ti–Au thin films

#### 3.1.1 Structural and chemical characterization

Results for chemical composition and thickness of the as-grown and ex-situ heat treated (ex-situ HT) Ti–Au thin films are shown in Table 1. These EDX results were taken from thin film samples deposited on soda lime glass substrates which are known to contain Si, O and other trace elements like Na, Mg, K and Ca, while Carbon is known for being a background error element in the EDX spectra [53,54]. Therefore, Table 1 contains the EDX results achieved after deconvoluting these background noise elements. For readers interested in EDX spectra of the deposited and heat treated thin films, please refer to Supplementary data 1. The film thickness was measured from cross-sectional SEM images (supplementary data 4-a) and it can be seen that the as-grown thin films are of thicknesses between 550 and 700 nm. The accuracy of film thickness was verified using a stylus profilometer and was within ±17 nm for all samples. After heat treatment, the thicknesses of the pure Ti (S_0.00_) and pure Au (S_1.00_) films are seen to be reduced drastically by over 100 nm, and this could be because of defect and void healing taking place at higher temperature [55,56]. A similar trend is observed for Ti rich films like (S_0.10_, S_0.20_), although the reduction from the as-grown thickness in these samples is smaller. With further addition of Au (S_0.24_ to S_0.82_), the film thickness remains stable around the as-grown values and could be attributed to a combined effect of grain growth of intermetallic phases like Ti_3_Au, TiAu, TiAu_2_ and TiAu_4_, plus increased oxidation of upper layers rather than defect healing [57].

**Table 1:** Chemical composition and thickness of Ti–Au thin films, as-grown and after ex-situ heat treatment in a tube furnace at 450 °C, [S.D = standard deviation within the EDX results].

Sample ID	As-grown			Ex-situ HT						
	Thickness (nm)	Ti composition (at%)[S.D]	Au composition (at%)[S.D]	Thickness total/new layer (nm)	Elemental composition (Ti: Au: O) in at%					
					Near Surface [S.D]			Mid Region [S.D]		
**S**_**0.00**_	691	100	0	567	45 [3.2]	00 [--]	55 [3.2]	61 [1.2]	00 [--]	39 [1.2]
**S**_**0.10**_	624	90 [0.09]	10 [0.09]	580**/**120	65 [1.9]	04 [1.3]	30 [1.9]	62 [1.4]	19 [1.3]	21 [0.5]
**S**_**0.20**_	561	80 [0.32]	20 [0.32]	513**/**110	38 [1.1]	02 [1.9]	60 [2.0]	65 [1.6]	21 [0.4]	14 [2.1]
**S**_**0.24**_	668	76 [0.05]	24 [0.05]	669**/**120	40 [1.4]	05 [1.9]	55 [1.9]	69 [1.6]	20 [1.4]	11 [0.5]
**S**_**0.28**_	621	72 [0.21]	28 [0.21]	645**/**118	49 [3.7]	15 [1.1]	36 [3.0]	63 [3.5]	18 [1.6]	19 [3.4]
**S**_**0.34**_	693	66 [0.27]	34 [0.27]	685**/**115	41 [2.9]	10 [2.3]	59 [3.2]	54 [2.8]	28 [2.4]	18 [2.5]
**S**_**0.53**_	636	47 [0.33]	53 [0.33]	673**/**91	30 [2.2]	02 [1.8]	68 [1.6]	31 [3.2]	50 [1.5]	19 [4.6]
**S**_**0.70**_	613	30 [0.22]	70 [0.22]	628**/**93	37 [1.6]	40 [1.7]	23 [1.9]	37 [1.9]	50 [0.8]	13 [0.9]
**S**_**0.82**_	630	18 [0.26]	82 [0.26]	668	34 [3.2]	04 [3.9]	62 [4.1]	16 [3.8]	74 [3.9]	10 [0.9]
**S**_**1.oo**_	554	0	100	450	00 [--]	100 []	00 []	00 [--]	100 []	00 []

**Fig. 3:**
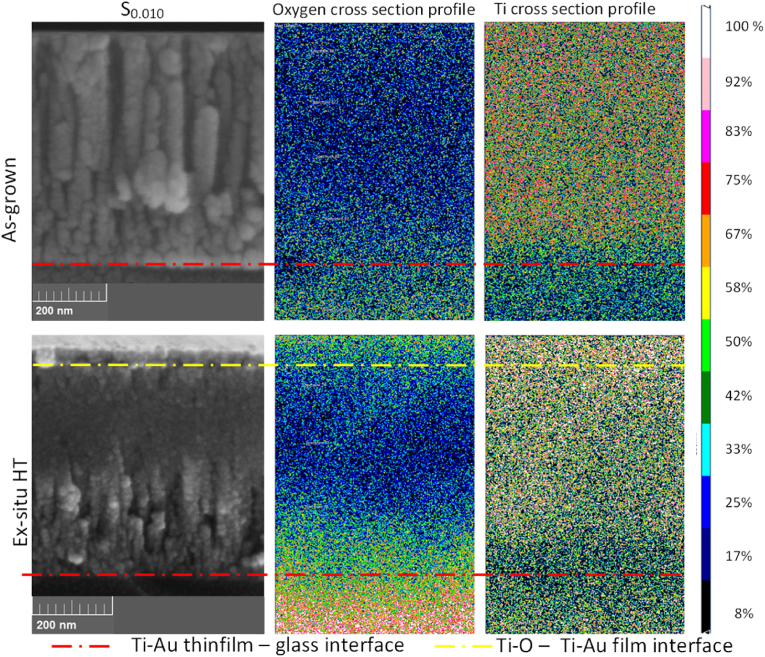
Low voltage EDX analysis of cross section area from S_0.10_ thin film, as-grown and following ex-situ heat treatment, showing heavy presence of oxygen on the top layers of the film surface.

The EDX results for chemical composition, taken from the average of five data measurement points at three different sites on the Ti–Au film surfaces, are also presented in Table 1. The ten Ti–Au thin film samples are identified according to their at% of Au and cover the entire spectrum of Ti–Au composition, to give a clearer picture of structural changes taking place with increasing Au concentration in the films. After heat treatment, the pure Ti film (S_0.00_) exhibits high levels of oxidation both in the surface layers and mid thickness regions due to the higher oxygen affinity of titanium [58,59]. With the addition of Au, EDX results show two distinctive regions: a surface layer which is comprised mainly of Ti and O, with very little Au in comparison and lower regions which are richer in Ti and Au, forming the intermetallics with less oxygen contamination. It can be argued that after heat treatment, Ti moves to the surface because of its higher affinity towards oxygen, and rearranges the Ti–Au composition in the lower region, leaving it richer in Au compared to its as grown state [57]. Only the pure Au film (S_1.00_) does not register any oxidation because of its lower reactivity towards oxygen [60]. This situation is further clarified by EDX mapping performed on the cross-section of as-grown and ex-situ HT S_0.10_ samples, see Fig. 3. The as-grown film shows an absence of oxygen and a uniform distribution of Ti throughout the film thickness. A clear contrast can also be seen between the TiAu film and the glass (SiO_2_) substrate underneath. However, following ex-situ heat treatment a strong presence of oxygen can be seen in the upper film layer (green spots) with a corresponding increase in Ti in the same region (white spots). Ti is not expected to react with silicon in the glass substrate to form silicide below 700 °C [61,62], and while its interaction with oxygen bounded to the glass-thin film interface begins around 400 °C as seen in the interface images below, it does not become significant until temperatures higher than 500 °C [63].

**Fig. 4:**
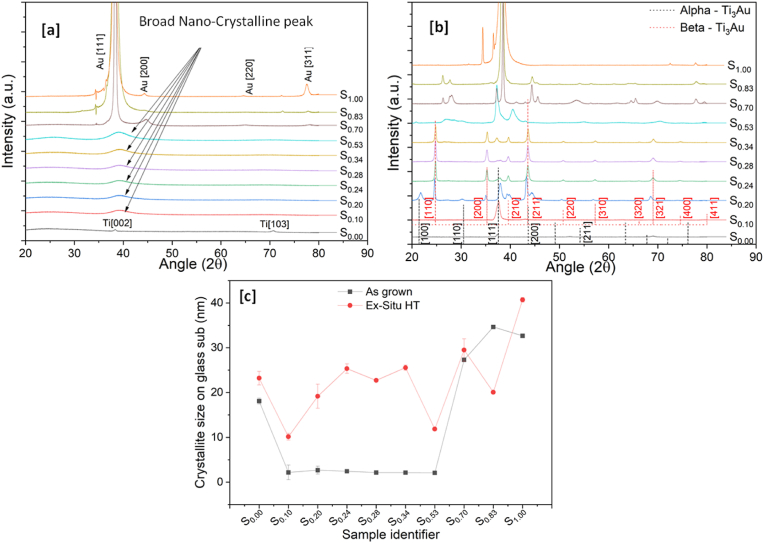
XRD patterns for (a) as-grown and (b) Ex-situ heat treated Ti–Au thin films deposited on glass substrates and (c) crystallite size calculated for these films.

The XRD patterns recorded for the as-grown thin films on glass substrates are shown in Fig. 4 (a). Pure Ti thin films are known to exist in three crystalline forms: α-phase, β-phase and γ-phase and when alloyed with Au form four intermetallics: Ti_3_Au, AuTi, Au_2_Ti, and Au_4_Ti with increasing concentration of Au in the film. The Ti_3_Au intermetallic exists in two different phases denoted as α-phase and β-phase. Both these phases are cubic structures, but the α-phase is formed at lower temperatures with an n°221 space group and Pm3 m symmetry, whereas the β-phase develops at temperatures above 350–400 °C with an n°223 space group and Pm3n symmetry. In the as-grown state, the pure Ti thin film (S_0.00_) exhibits two clear peaks at 38.5° and 70.7°, confirming the presence of Ti in the α-phase (Fig. 4a). However, with addition of Au in the Ti matrix, none of the film compositions show a clearly developed crystalline structure until sample S_0.70_. In the Ti rich zone (S_0.10_, S_0.20_, S_0.24_, S_0.28_, S_0.34_) and at equi-atomic concentration (S_0.53_), a very broad peak can be seen developing between 36° and 42° with peak position around 39°. Most of the Ti–Au intermetallics have at least one strong peak located in this region, like the characteristic (210) plane of β-Ti_3_Au at 39.6° or that of β-TiAu at 38.9°. Similar broad peaks, indicate a quasi-crystalline microstructure and/or a mixture of various phases of different TiAu intermetallics. With a further increase in Au concentration in the films (S_0.70_ and S_0.83_), very strong characteristic Au peaks develop at 38.3° and 44.7°. While the peak at 44.4° indicates the presence of Au, the larger angular width of the peak at 38.3° could possibly be overlapping the quasi-crystalline Ti–Au intermetallic specific peak present around the same angular point. The pure Au film (S_1.00_) exists in the face centred cubic structure with Fm3m symmetry as identified by its characteristic peaks at 38.3°, 44.4°, 64.6°, and 77.6°. The XRD spectrums from Ti–Au thin films grown on Ti_6_Al_4_V substrates were found to match very well with the results on glass substrates and are available in the supplementary section (data 2).

An external source of thermal energy like heat treatment or substrate heating raises the ad-atom mobility to move towards the lattice site and thereby improves the films crystallinity. Therefore, the ex-situ heat treatment of as-grown films at 450 °C, will lead to better crystallization of Ti–Au intermetallic phases and thereby segregate the broad quasi-crystalline peaks into characteristic diffraction peaks, enabling accurate and reliable identification of these phases. Fig. 4b shows the XRD patterns achieved for Ti–Au thin films grown on glass substrates after heat treatment at 450 °C inside a tube furnace in a sealed Ar environment (ex-situ HT). The central peak positions of the Ti _3_Au intermetallic α and β-phases are also shown in red and black dashed lines in Fig. 4b along with the corresponding orientation for reference. With heat treatment, the peak positions for the pure Ti film (S_0.00_) shift to lower angles of 38.2° and 69.1°, indicating a change from the α to β-phase of Ti. However, the most dramatic change in structure is observed for the Au doped films (S_0.10_, S_0.20_, S_0.24_, S_0.28_, S_0.34_, and S_0.53_). The broad quasi-crystalline peak observed in the as-grown state is replaced by well-defined and identifiable peaks of various Ti–Au intermetallics following ex-situ heat treatment. For S_0.10_, a clear peak located at 37.5° indicates development of the α-phase of the Ti_3_Au intermetallic, with strong preferential orientation along the (111) direction and no other peaks for α or β-phase exist. With further addition of Au (S_0.20_) a mixture of α and β-phases emerges. Clear α peaks exist at 21.4° and 30.4°, representing the (100) and (110) orientations, while the peak at 37.9° representing the (111) orientation, appears to be under strain as it is also transitioning towards the β-phase. There are clear β peaks at 24.7°, 35.1°, 39.4°, representing the (110), (200) and (210) orientations respectively and a strained peak at 43.1° representing the (211) orientation. WPPF refinement quantifies this sample to contain 86.5 wt% of α-phase against 13.5 wt% of β-phase. Therefore, the results clearly show that transition of α into β-phase takes place with increased temperature at appropriate Au concentration. When the Au concentration reaches ∼25 at% (S_0.24_) the peak positions at 24.7°, 35.2°, 39.6°, 43.6°, 50.7°, 57.3°, and 68.9° align very well with the peak positions representing the (110), (200), (210), (211), (220), (310), (321) orientations of the β-phase respectively. One peak positioned at 37.6°, representative of the (111) orientation of the α-phase, is also present. For this sample, WPPF refinement calculates 83.8 wt% of the sample to be in β-phase with only 16.2 α-phase present. This finding shows that in order to achieve the β-Ti_3_Au intermetallic with well oriented crystallites, it is essential to fine tune the chemical composition and thermal treatment temperature during deposition. For the next sample (S_0.28_), the diffraction pattern is almost identical to that of S_0.24_, which is also reflected in the calculated α:β ratio of 18:82 wt% obtained from phase quantification. The small increase of 4 at% Au will only cause very small changes in the film microstructure which may be outside the detection limit of the XRD instrument [64,65] With further addition of Au in the film S_0.34_, the β-phase stability is lost, and the α-phase starts to emerge again with a peak at 37.4°, representing the (111) orientation of α-Ti_3_Au. The intensity of the peak representing the β-phase is reduced compared to that for the S_0.24_ or S_0.28_ thin films. Peaks corresponding to Au_2_Ti and AuTi also begin to emerge in the curve fitting and the final weight distribution calculated from phase analysis is α-Ti_3_Au – 65 wt%, β-Ti_3_Au – 23.3 wt%, AuTi- 9.4 wt% and Au_2_Ti – 2.3 wt%. Thereafter, with further increase in Au concentration the Ti_3_Au intermetallic is replaced by other intermetallics of the Ti–Au alloy system, for example S_0.53_ is seen to be a mixture of TiAu (peaks positioned at 40.3°, 55.1°, and 70.4°) and TiAu_2_ (peaks positioned at 20.7° and 37.3°), while S_0.70_ appears to be a mixture of TiAu_2_ (peaks positioned at 28.3°, 41.4°, 53.4°, 65.5°, 70.1°) and TiAu_4_ (peaks positioned at 26.3°, 27.6°, 43.0°, 53.4°, 56.3°, 64.4°, 77.8°). Quantitative analyses of the pattern shows that S_0.53_ contains 66.5 wt% of AuTi and 14.6 wt% Au_2_Ti, while the presence of Ti_3_Au phases reduces to 18.9 wt%. Thereafter, the next sample S_0.83_ forms TiAu_4_ intermetallics (peaks positioned at 26.3°,27.6°, 44.3°, 77.8°). Finally, the pure Au thin film (S_1.00_) develops preferential orientation along the (111) direction at 38.3° with a small reflection peak at 77.6° for the (311) direction. No oxides of Ti are observed in the XRD patterns because TiO_2_ is amorphous at room temperature and begins to form the anatase phase above 400 °C and rutile at much higher temperature, but with addition of alloying elements like Au, this temperature increases beyond that of 450 °C used in this study [66,67]. Overall, the XRD patterns show that Ti rich Ti–Au thin films grown at room temperature are quasi-crystalline in nature but when sufficient thermal energy is provided to the system from ex-situ heat treatment, the structure perfection improves dramatically, such that the characteristic peaks of individual Ti–Au intermetallics start to appear at their respective characteristic peak positions and can be quantified by the reduction in fullwidth at half maximum (FWHM) of the corresponding diffraction peaks [[68], [69], [70]]. This is very clearly visible for Ti rich zones (S_0.10_, S_0.20_, S_0.24_, S_0.28_, S_0.34_), as these samples register a 5 to 11 times reduction in FWHM after ex-situ heat treatment, when compared to their as-grown counterparts. According to Svanidze et al., together with structure perfection, the presence of minority phases and defects like α-Ti_3_Au or distributed oxides, may interact favourably with the majority β-phase to further inhibit the dislocation motion within the lattice leading to enhancement of mechanical hardness of the coating [28]. And ex-situ heat treatment together with better crystallinity and FWHM reduction, also leads to inclusion of oxides in the thin film structure. Therefore, it can be agreed that structural perfection of the β-phase can be improved by ex-situ heat treatment while the ratio of α to β phases can be achieved by maintaining the correct 3:1 ratio of Ti:Au in the growing film during deposition.

Fig. 4c presents the crystallite size of the TiAu thin film samples calculated from the leading peak in the diffraction pattern, in both as-grown and ex-situ heat treated states. In the as-grown state the pure Ti thin film (S_0.00_) is calculated to have a crystallite size of 18 nm which is reduced to values lower than 3 nm for samples S_0.10_, S_0.20_, S_0.24_, S_0.28_, S_0.34_ and S_0.53_, based on the broad quasi-crystalline peaks seen for these films. Crystallites of the Au rich films, S_0.70_, S_0.83_ and S_1.00_ are calculated to have sizes of 27, 34 and 32 nm respectively, and are again expected because of the extremely sharp Au peaks observed for these compositions. With heat treatment, the pure Ti film (S_0.00_) changes from α to β-phase thereby registering a slight increment in crystallite size to 23 nm. The rest of the Ti rich films also register increases in their calculated crystallite sizes of 10, 19, 25, 22, 25 and 11 nm for S_0.10_, S_0.20_, S_0.24_, S_0.28_, S_0.34_ and S_0.53_ respectively. This could be corelated to development of Ti–Au intermetallic compounds post heat treatment. The Au rich film S_0.70_ only shows a slight increment in crystallite size to 29 nm while S_0.83_ shows a sudden decrease to 20 nm. This could be explained by the separation of newly developed Ti–Au intermetallic peaks in these samples after heat treatment, whereas previously in the as-grown state, they were merged with the Au peak, giving larger crystallite sizes. Finally the pure Au thin film (S_1.00_) shows an increase of 8 nm to take the crystallite size to 40 nm after heat treatment because of the preferential orientation of Au improving along the (111) direction. Phase transformation, defect healing and grain growth caused by heat treatment also aids relaxation of internal forces confined within the Ti–Au thin films during the deposition process [[71], [72], [73]] and this relaxation has been quantitatively measured by Scherrer's method [ε = βτ/4tan(θ)] for all thin films and by the Williamson-Hall plot method for better crystallized heat treated thin films [74]. For interested readers these results are provided in the supplementary section (data 3). But, it is very clear now that the room temperature deposited quasi-crystalline Ti–Au thin films achieve better crystallization, phase transformation, structure perfection and stress relaxation leading to the emergence of superior quality Ti–Au intermetallics after ex-situ heat treatment.

#### 3.1.2 Morphology characterization

**Fig. 5:**
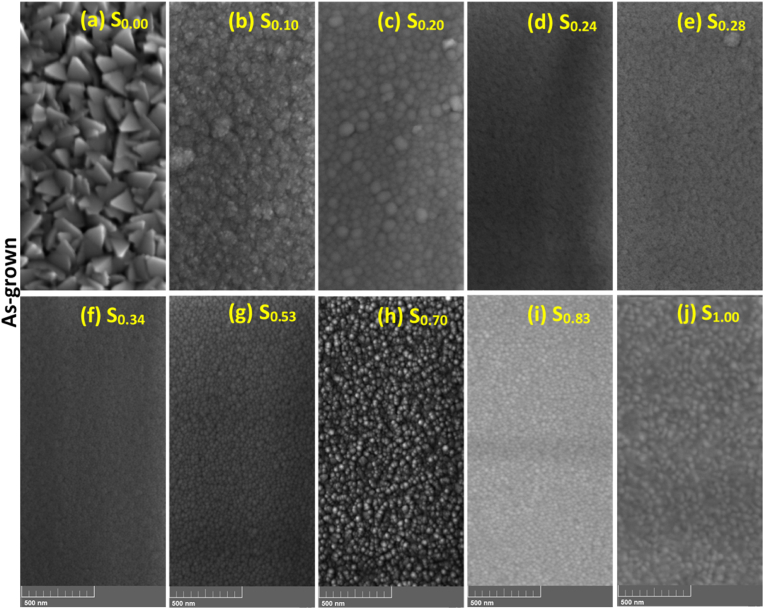
Surface SEM images of as-grown Ti–Au thin films deposited on glass substrates with increasing Au composition.

Surface images showing the effect of increasing Au concentration on the grain structure of the as-grown Ti–Au thin films are presented in Fig. 5 (a to j). The as-grown pure Ti thin film, S_0.00_ (Fig. 5a) has a clear triangular shaped morphology with individual grains, roughly 160 ± 12 nm long. But with addition of Au, the grain shape and size drastically change to circular clusters of around 84 ± 8 nm for S_0.10_ (Fig. 5b) and then to well defined circular shape grains of 54 ± 25 nm for S_0.20_ (Fig. 5c). This change in grain shape and size, corelates very well with the XRD patterns seen in Fig. 4 (a), where crystalline peaks of Ti disappear, and are replaced by broad peaks representing nano-crystalline Ti–Au intermetallics. Further additions of Au (S_0.24_, S_0.28_ and S_0.34_) leads to the development of extremely fine Ti–Au alloy grains, which are not clearly distinguishable in Fig. 5 (d), (e) and (f) and therefore correlate well with the nano-crystalline nature of the Ti–Au intermetallic observed in the XRD patterns. For sample S_0.53_ (Fig. 5g) the grains appear to grow larger compared to previous Ti rich samples, although these grains are still seen to be nano-crystalline in the corresponding XRD pattern. With further addition of Au, the surface appears coarser with the continued growth of circular grains for S_0.70_ and S_0.83_ (Fig. 5h–i), a condition supported by the development of sharp Au dominated peaks in the XRD pattern. Finally for the pure Au thin film S_0.10_ (Fig. 5j), the grains are circular with sizes of around 45 ± 8 nm.

**Fig. 6:**
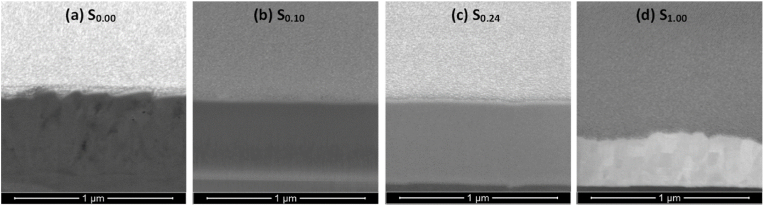
Focussed ion beam (FIB) cross section images of as-grown Ti–Au thin films (a) S_0.00_ (b) S_0.10_, (c) S_0.24_, and (d) S_1.00_ deposited on Ti_6_Al_4_V substrates.

These results are further supported by the selective cross sectional FIB images presented in Fig. 6(a–d), where thin film samples S_0.10_ and S_0.24_ were selected for their superior hardness results (see section 3.1.3) and the pure Ti sample S_0.00_ and pure Au sample S_1.00_ were selected as references from either extreme of the Ti–Au composition spectrum. The cross section of the as-grown pure Ti film (Fig. 6a) shows large polyhedral grains, similar to those seen in the surface SEM image in Fig. 5a. The cross section of the as-grown S_0.10_ sample (Fig. 6b) shows development of very fine grain columnar structure, which becomes very dense and completely featureless for sample S_0.24_ (Fig. 6c). These results are in good agreement with the surface SEM images (Fig. 5b and d) and their corresponding XRD patterns (Fig. 4) and suggest that addition of Au in the Ti matrix leads to formation of an extremely dense and fine-grained structure. On the other hand, the pure Au thin film S_1.00_, although thinner in film thickness, develops very large grain sizes (Fig. 6d).

**Fig. 7:**
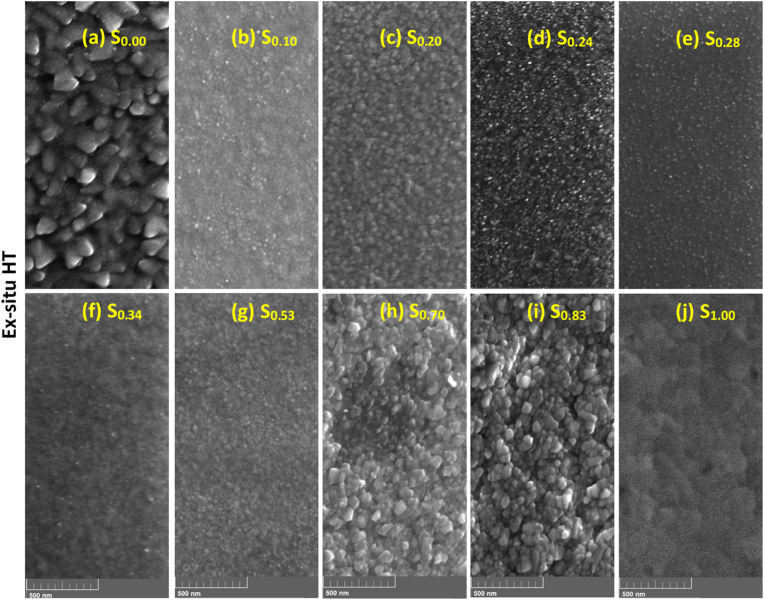
Cross-section SEM images of Ex-situ heat treated Ti–Au thin films (a) S_0.00_ (b) S_0.10_ and (c) S_0.24_, deposited on Ti_6_Al_4_V substrates after FIB polishing.

The effect of ex-situ heat treatment on the surface morphology of the Ti–Au thin film samples is shown in Fig. 7 (a to j). As evidenced from the previous surface and cross-section EDX results, heat treatment leads to surface oxidation of the films and therefore the surface images seen here consist predominantly of titanium oxide intermixed with Ti–Au intermetallics. Fig. 7a shows that the polyhedral shaped grains seen in the as-grown pure Ti thin film, S_0.00_ (Fig. 5a), fuse together after heat-treatment and become oval shaped. The Ti rich films (S_0.10_, S_0.20_) show a decrease in grain size as the circular grains become more densely packed (Fig. 7b and c). But films which exhibited extremely fine grains in the as-grown state, like S_0.24_. S_0.28_ and S_0.34_ (Fig. 7d–f), begin to grow needle like structures after heat treatment, with more intense growth for films with higher Ti concentration. Following heat treatment, the α and β-phases of the Ti_3_Au intermetallic begin to emerge in these Ti rich compositions, giving them a similar visual appearance, which agrees well with the similar XRD patterns observed for these films in Fig. 4. As the Ti–Au concentration reaches a ratio of approximately 1:1 for S_0.53_ (Fig. 7g), the film starts to develop a densely packed grain structure of random shapes. For Au rich films, S_0.70_ and S_0.83_ (Fig. 7h and i) the grains appear to have grown in size compared to their as-grown counterparts. The circular grains observed for the pure Au thin film S_1.00_ in the as-grown state, rapidly grow in size after heat treatment, but their growth is random in shape and appears very flat (Fig. 7j) when compared to the raised 3D grains observed in the S_0.70_ and S_0.83_ films.

**Fig. 8:**
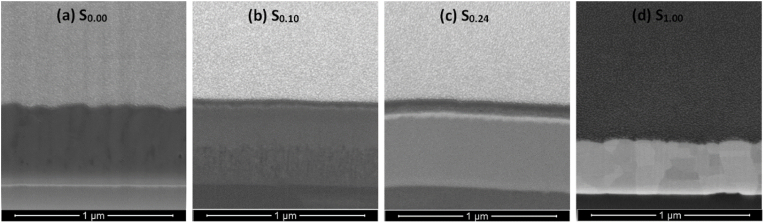
Focussed ion beam (FIB) cross section images of ex-situ heat treated Ti–Au thin films (a) S_0.00_ (b) S_0.10_, (c) S_0.24_, and (d) S_1.00_ deposited on Ti_6_Al_4_V substrates.

Although Fig. 7 summarizes the film surfaces after heat treatment, the effect on the formation of oxides in the upper layers of the Ti–Au intermetallics is not clearly shown. To this end, selective cross-section FIB images of Ti–Au thin films S_0.00_, S_0.10_, S_0.24_ and S_1.00_ following heat treatment are shown in Fig. 8 (a to d). Increased surface diffusion caused by heat treatment leads to densification of the thin films from their as-grown state and can enhance mechanical hardness. Specifically, the pure Ti thin film S_0.00_, gains a densely packed columnar structure after heat treatment (Fig. 8a), as formation of amorphous titanium oxide is known to exhibit a very dense cross section with no grains or clusters [75,76]. Moreover, sample S_0.10_ (Fig. 8b) develops a partial columnar structure after heat treatment while the S_0.24_ film (Fig. 8c) remains extremely dense with virtually no grain structure. It can be noted that after heat treatment most of the Ti rich thin films form a secondary layer on their upper surface, consisting primarily of titanium oxide. The chemical composition and thickness of this oxide layer is shown in Table 1. While the FIB image of the externally heat treated pure Au film (Fig. 8d) also shows better grain growth taking place with heat treatment, the oxide layer is absent in this sample due to the low affinity of Au towards oxygen [77]. Therefore, the change in grain structure of the Ti–Au intermetallic thin films and the growth of the surface oxide layer is very well established from these cross-sectional images.

**Fig. 9:**
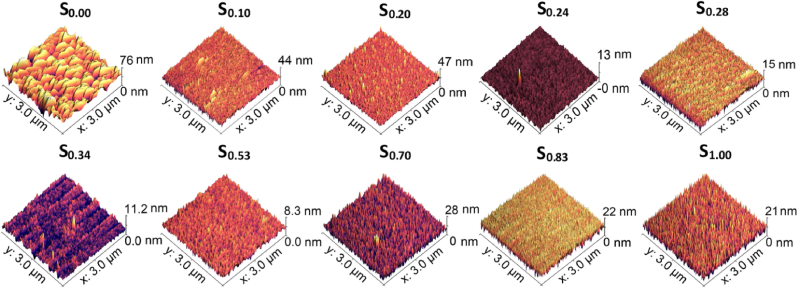
AFM micrographs of as-grown Ti–Au thin films deposited on glass substrates with increasing Au composition.

Even though the SEM images provide a visual representation of the thin film surfaces, because of the extremely small feature sizes of the Ti–Au intermetallics in the as-grown state and titanium oxides in the heat-treated state, a surface probing technique is required to achieve a quantitative estimation of the morphological features on the thin film surfaces. Surface characteristics of the as-grown thin films were recorded using an AFM with a silicone tip in contact mode and the resulting 3D micrographs are shown in Fig. 9. The vertical scale bars give an indication of the highest feature measured on a 3 by 3 μm area on each film sample. The as-grown pure Ti thin film (S_0.00_) is observed to have uniformly distributed triangular grains with a maximum feature size of 76 nm. Ti rich films (S_0.10_ and S_0.20_) present thick needle shaped grains with sizes in the range of 45–50 nm. Similar to the SEM results (Fig. 5), further addition of Au leads to a drastic reduction in the height of the needle shaped grains to values below 15 nm for the S_0.24_, S_0.28_, S_0.34_ and S_0.53_ films. This result aligns very well with the XRD patterns observed in Fig. 4, which show poor crystallization of the Ti–Au intermetallic in the as-grown state. The Au rich films such as S_0.70_, S_0.83_ and S_1.00_, exhibit a similar needle like structure but with increased height range above 20 nm, due to the domination of Au crystallization, as observed in the XRD and SEM results.

**Fig. 10:**
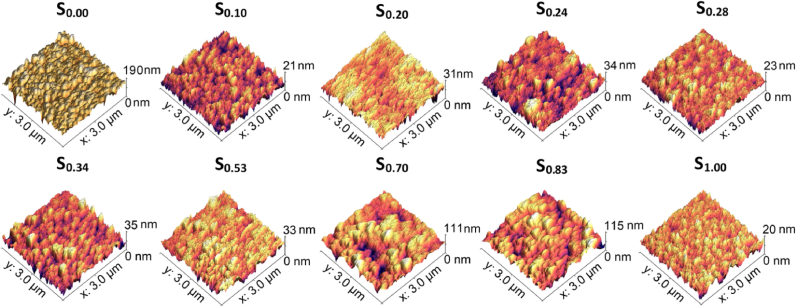
AFM micrographs of ex-situ heat treated Ti–Au thin films deposited on glass substrates with increasing Au composition.

AFM 3D micrographs of the ex-situ heat treated Ti–Au thin films are shown in Fig. 10. The pure Ti thin film (S_0.00_) shows a steep increment in surface feature height following heat treatment, with hill shaped structures of up to 190 nm. This drastic change could be attributed to a combined effect of grain growth and surface oxidation. The Ti rich samples (S_0.10_ and S_0.20_) show merging dome shaped structures with a reduction in feature size to 21 and 31 nm, respectively. Whereas the other Ti rich samples (S_0.24_, S_0.28_, S_0.34_ and S_0.53_) show a relative increase in feature heights following heat treatment, with values in the range of 23–35 nm. These results are in accordance with the SEM images shown in Fig. 7 for thin films with 10–34 at% Au content and strengthen the theory that various ratios of α and β-Ti_3_Au intermetallic are developed with heat treatment. The Au rich samples (S_0.70_ and S_0.83_) show raised structures with well curved peaks and a very steep increase in their feature sizes to values of 110–115 nm after heat treatment. In contrast the pure Au thin film (S_1.00_) exhibits sharper dome shaped grains reaching feature sizes of only 20 nm, which corresponds with the relatively flat surface observed in the SEM image of this film in Fig. 7.

Surface roughness is another quantity which can be gained from analysing the AFM images to give a better estimation of surface conditions. Table 2 summarizes the results for the Ti–Ag thin film samples in their as-grown and ex-situ heat treated states (results for in-situ Tsub films will be discussed later). The surface roughness of the pure Ti thin film (S_0.00_) increases from 9.7 to 15.9 nm following heat treatment. This result is supported by the SEM and XRD data and suggests that the film undergoes grain growth and heavy oxidation during heat treatment, resulting in rougher surface features. Compared to the pure Ti thin film, the as-grown Ti–Au (S_0.10_ to S_0.53_) films exhibit a reduction in surface roughness from 3.3 to 0.6 nm, expected from the sudden change in film topography seen in the SEM images due to the formation of quasi-crystalline Ti–Au intermetallics. The surface roughness of the as-grown films starts to increase again to around 2.5 nm from samples S_0.70_, as increasing Au concentration leads to the emergence of Au crystalline phases. However, after heat treatment, the surface roughness stabilizes to values between 2.6 and 4.4 nm for samples S_0.10_ to S_0.34_, due to the emergence of a mixture of α and β-phases of Ti_3_Au intermetallic for this composition range. Surface roughness increases briefly for S_0.70_ and S_0.83_–14 nm, before reducing again to 2.1 nm for the pure Au thin film (S_0.00_). When analysed together with the SEM results seen earlier, it can be seen that the formation of a Ti–Au quasi-crystalline structure drastically reduces the surface roughness when compared to pure Ti thin films. And while external heat treatment was observed to have a strong effect on crystal structure, due to improved crystallization of Ti–Au intermetallics, the thermal activation also leads to a subtle increase in the surface roughness of heat treated thin films, which could be attributed to surface oxidation and grain growth [78,79].

**Table 2:** RMS surface roughness of Ti–Au thin films measured from AFM micrographs for as-grown, ex-situ heat treated and films deposited with in-situ substrate temperature.

Test Sample	RMS roughness (nm)		
	As-grown	Ex-situ HT	In-situ Tsub
S_0.00_	9.7	15.9	7.2
S_0.10_	3.3	2.6	6.5
S_0.20_	2.9	3.4	4.4
S_0.24_	0.6	4.4	1.6
S_0.28_	1.6	2.4	1.2
S_0.34_	0.8	4.4	–
S_0.53_	0.6	3.8	–
S_0.70_	2.8	14.1	–
S_0.83_	2.3	14.6	–
S_1.00_	2.5	2.1	–

#### 3.1.3 Mechanical characterization

**Fig. 11:**
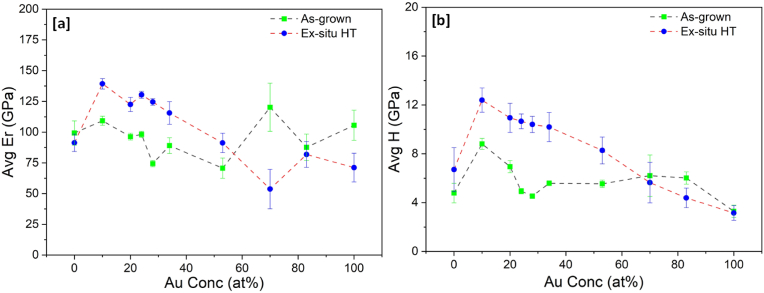
Variation of (a) Reduced elastic modulus (Er) and (b) Hardness (H) of as-grown and ex-situ heat treated Ti–Au thin films, deposited on glass substrates with increasing Au composition.

Mechanical properties of reduced elastic modulus and hardness, extracted from Ti–Au thin films deposited on glass substrates using the nanoindentation technique, are shown in Fig. 11 (a) and (b) respectively. Reduced elastic modulus takes into consideration the elastic deformation caused on both the sample under test and the indenter used, without the need to include the Poisson's ratio for each of the materials, thereby simplifying the analysis of the results [80]. The reduced elastic modulus of the pure Ti and Au thin films in the as-grown state is found to be 99 ± 9 GPa and 105 ± 10 GPa, respectively. A similar increment in the elastic modulus of Au thin films has been justified by the dominant role of intrinsic stresses when film dimensions change from bulk to microscale [81,82]. In contrast, for Ti–Au alloys, the as-grown thin films show a decreasing trend in elastic modulus with increasing concentration of Au in the films, with the exception of S_0.70_, which shows an unexpected peak of 120 GPa. Among the Ti rich films, the highest value of 109 GPa is achieved for S_0.10_. However, after heat treatment the trend for elastic modulus becomes smoother with a sudden increase from 91 GPa for the pure Ti film, to a peak value of 139 ± 4 GPa for S_0.10_ and thereafter decreases with increment in Au concentration, until reaching a value of 71 GPa for the pure Au film. It is known from the XRD results that the as-grown thin films are in quasi-crystalline state with various coexisting Ti–Au intermetallics, which results in variations in the reported elastic modulus values. However, after heat treatment, the various distinguished Ti–Au phases develop and give rise to a smoother and more predictable trend in the elastic modulus curve.

Mechanical hardness for the as-grown pure Ti thin films is measured to be 4.7 ± 0.8 GPa and increases to a moderate hardness value of 8.8 ± 0.4 GPa with addition of 10 at% Au in the film (S_0.10_). Thereafter, with further increases in Au concentration in the films, the hardness value slowly decreases, staying in the range of 4.5–6.9 GPa for rest of the Ti–Au composition range, with the pure Au thin film having a hardness of 3.2 ± 0.5 GPa. For lower Au concentration regions, the low energy of atoms sputtered at room temperature leads to diffusion of metallic Au ions into the Ti structure acting as defects and because of the quasi-crystalline nature of Ti mixed with Au, there is uniform distribution of these defects [80]. This leads to the hardness improvement seen for S_0.10_ with addition of Au, as the increase in defects presents a higher dislocation barrier within the amorphous structure. However, for Au concentrations above 15 at%, a mixture of Ti–Au intermetallic compounds begins to develop, as indicated by the broad peaks in the XRD patterns, which weakens the overall film structure [83,84]. It is well known, that thin films can be strengthened by increasing the dislocation energy barrier which can be achieved by increasing the density of point/line defects or by increasing the internal boundaries via grain size control, phase development or column densification or by increasing the number of solute/secondary phase particles within the alloy matrix [80,85,86]. All these phenomena can coexist and have a combined effect on the hardness value of thin films. After heat treatment, the value of hardness of the pure Ti thin film increases to 6.7 ± 1 GPa, which can be explained by the development of the β-phase of Ti, which acts as a secondary phase particle in the Ti matrix. Thereafter, with further addition of Au, a very steep increase in hardness is observed to 12.3 ± 0.9 GPa for the S_0.10_ film before steadily decreasing to a value of 3.1 ± 0.6 GPa for the pure Au thin film. This trend can be correlated to two factors: (i) development of the super hard Ti_3_Au phase with dense atomic coordination, resulting in shortened atomic bonds and high hardness, and (ii) the oxidation of the film which gives rise to an increased number of defects that act as barriers to dislocation movement in the thin film. On the other hand, there is also an effect from grain growth which leads to reduction in grain boundaries thereby causing a decrease in hardness values [87]. From the previous work it is expected that hardness will peak at the 75:25 at% ratio of Ti:Au due to the formation of β-Ti_3_Au at this composition range [9,28,80]. However, in this work the hardness is observed to peak for sample S_0.10_, with a Ti:Au ratio of 90:10 at%. From the cross-sectional EDX images (Fig. 3) it is clear that heat treatment causes a migration of Ti to the upper film layers to react with oxygen, leaving the lower regions richer in Ti. This rearrangement of the Ti:Au composition leads to ideal conditions for formation of the preferentially ordered α-Ti_3_Au intermetallic in the lower regions of the S_0.10_ sample, as evidenced by the uniform columnar structures seen in the cross-section FIB image of this film (Fig. 8b). The calculated crystallite sizes in Fig. 4 also support the improvement of this Ti_3_Au intermetallic phase, while the SEM and AFM images show that the corresponding grain size growth is not as large for other Ti rich samples. All these factors, combined with intermixed titanium oxides in the film surface, lead to enhanced resistance to dislocation propagation and increase the hardness of the S_0.10_ sample, even in absence of the β-phase of Ti_3_Au. However, with further addition of Au in the film (S_0.20_, S_0.24_, S_0.28_ and S_0.34_), the rearranged composition after migration of Ti caused by heat treatment, leads to development of a mixture of α and β-phases, instead of a preferential single-phase development as seen for sample S_0.10_. This is evidenced by the peak positions in the XRD patterns, weight distribution for different phases calculated using WPPF analysis and supported by the absence of well-defined structures in the lower regions of the film cross sections (see supplementary data 4b). From the AFM surface scans, it can be seen that these samples also depict larger grain growth when compared to the S_0.10_ sample, and therefore a steady reduction in grain boundaries aids the steady decline in hardness according to the Hall-Petch relationship [88]. Therefore it can be summarized that an enhanced hardening effect of the Ti–Au solid solution can be observed with formation of super hard Ti_3_Au intermetallic phases, improving growth defects such as oxides, reduction in grain size and densification of grain boundaries, which can be achieved by accurate tuning of atomic composition and heat treatment conditions of these Ti–Au thin films [80].

**Fig. 12:**
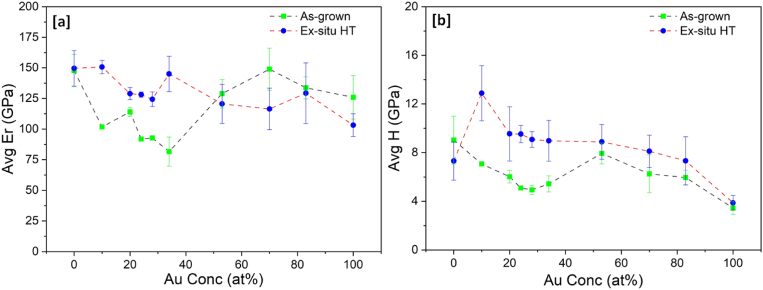
Variation of (a) Reduced elastic modulus (Er) and (b) Hardness (H) of as-grown and ex-situ heat treated Ti–Au thin films deposited on Ti_6_Al_4_V substrates with increasing Au composition.

The results of reduced elastic modulus and mechanical hardness of the Ti–Au thin films deposited on Ti_6_Al_4_V substrates are shown in Fig. 12 (a) and (b), respectively. The trend for elastic modulus for as-grown thin films on Ti_6_Al_4_V substrates is similar to that for glass substrates but with slightly higher values. The pure Ti films have an elastic modulus of 147 ± 13 GPa, which decreases from 101 to 81 GPa with addition of Au for samples S_0.10_ to S_0.34_. Thereafter, the elastic modulus increases again to values between 128 and 148 GPa for samples S_0.53_, S_0.70_ and S_0.83_, before reducing back to 125 ± 15 GPa for the pure Au thin films. After heat treatment, the Ti rich samples (S_0.10_, S_0.20_, S_0.24_, S_0.28_ and S_0.34_) show an increase in elastic modulus compared to their as-grown state, while Au and Au rich samples (S_0.53_, S_1.00_) show a decreasing trend. The reason for the increased values can be attributed to the higher elastic modulus of 114 GPa for the underlying Ti_6_Al_4_V substrate compared to that of glass which registers only 73 GPa [89,90]. Elastic modulus has a longer field of interaction compared to hardness which means it can be easily affected by the stiffness of the underlying substrate [90]. Even though the elastic modulus increases for Ti–Au films deposited on Ti_6_Al_4_V substrates, the results are still much lower than the value of 200 GPa commonly observed for Ti–Au intermetallics [9,80]. It can be argued that the extremely high elastic modulus of the Si substrates (172 GPa) used to deposit these films on, could have contributed to the high elastic modulus values observed in previous work [90]. The low elastic modulus values of the Ti_3_Au thin film coatings on Ti_6_Al_4_V substrates is a very good indicator that this material combination could be used in developing long lasting knee and hip implants to help to overcome the stress shielding effect that leads to poor bone re-growth and ultimately to reconstruction surgery [91].

Similar to their glass counterparts, the results for TiAu film hardness on Ti_6_Al_4_V substrates show a much clearer trend (Fig. 12 b), depicting a sharp peak of 12.8 ± 2 GPa for the heat treated S_0.10_ samples, while pure Ti and all the other Ti rich samples (S_0.20_, S_0.24_, S_0.28_ and S_0.34_) register values around 9 GPa. The hardness values continue to decrease for the Au rich films, with the pure Au films achieving 3.9 ± 0.6 GPa. While the trends for mechanical properties of Ti–Au thin films deposited on Ti_6_Al_4_V are very similar to those on glass substrates, the deviation of results (error bars) are seen to increase by a factor of 2–3. One key difference between the two substrate types is their surface finish. Even though the Ti_6_Al_4_V substrates are polished rigorously to achieve a surface roughness of <40 nm, they are still much rougher than glass, which registers surface roughness values of <2 nm. Previous simulations and experimental works have shown that increasing surface roughness leads to an increase in the average hardness values and the scatter of measurements made by the nanoindentation technique using a Berkovich tip [[92], [93], [94], [95]].

Previous studies have also suggested that the underlying substrate (glass, metalloid (Si etc.) or metallic) can also affect the thin film growth mechanism, propelled by differences in their surface features, which act as favourable nucleation sites during thin film growth, leading to stark differences in grain shape and size, and in turn the resulting coating properties [96]. However, the effect of film growth mechanisms caused by surface features such as nucleation sites, starts to diminish at film thickness greater 150 nm as the crystal grains begin to grow laterally rather than vertically, due to relaxation of stress, caused by increasing film thickness [97,98]. This change in growth mode with increasing film thickness is accompanied by film densification, improved crystallization, crystallite size increment, defect healing, and reduction in substrate-to-thin film strain misfit [98]. Therefore, in this work, the variation in mechanical properties of the relatively thick (∼600 nm) TiAu thin films are more strongly influence by the increased elastic modulus and surface roughness of the Ti_6_Al_4_V substrates compared to the glass substrates [90], than by substrate driven thin film growth mechanisms. Regardless of these minor differences, the results still show that Ti–Au thin films can develop super hardness values with lower elastic modulus on both glass and Ti_6_Al_4_V substrates, with proper control of chemical composition and heat treatment protocols.

#### 3.1.4 Cytotoxicity-biocompatibility tests on L929 cells

**Fig. 13:**
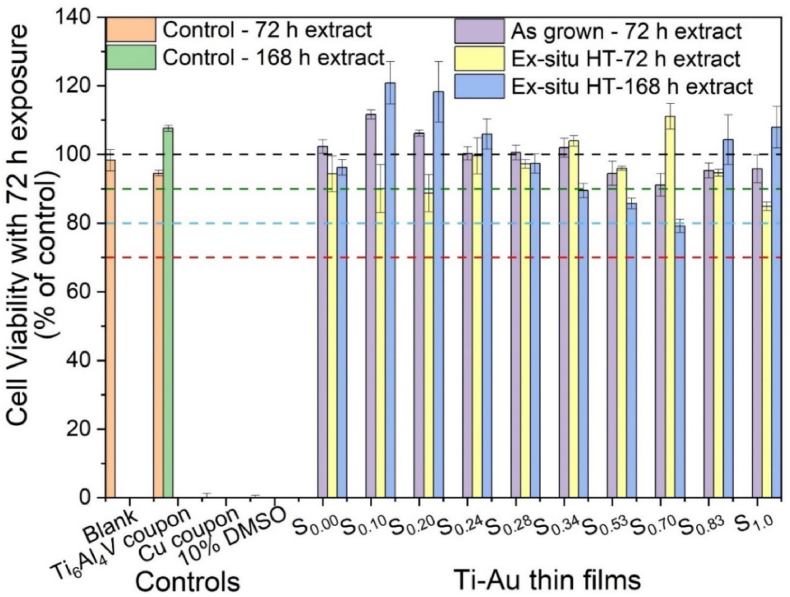
Cytotoxic effect on L929 cells following 72 h of exposure to 72 h and 168 h extracts from Ti–Au thin films in their as-grown and ex-situ heat treated states, compared to negative controls (untreated (BLANK) and Ti_6_Al_4_V coupon) and positive controls (Cu coupon and 10% DMSO).

In order to evaluate the biological interaction and the cytotoxic effect of different Ti–Au thin film derived extracts, the Alamar Blue assay was conducted on L929 mouse fibroblast cells. Specifically, L929 cells were incubated for 72 h with DMEM culture media containing leached anions from ten different Ti–Au thin films (S_0.00_, S_0.10,_ S_0.20,_ S_0.24_ S_0.28,_ S_0.34_, S_0.53,_ S_0.70,_ S_0.83_ and S_1.00_) following 72 h and 168 h of extraction, as described in Section 2.4. Moreover, L929 cells were treated with pure DMEM media (BLANK) and leached media from Ti_6_Al_4_V coupons (both considered as negative controls). Similarly, cells were also exposed to DMEM culture media containing anions derived from Cu coupons, as well as to 10% of DMSO (both considered as positive controls). It is worth mentioning that in order to observe the maximum effect on L929 cells' viability, undiluted DMEM anion-leached media was used in our experiments for all tested samples. According to data analysis (Fig. 13), all tested Ti–Au thin film derived extracts (both as-grown and ex-situ HT) were found to possess a safe cytotoxic profile, following 72 h incubation of L929 cells, as their effect on cell viability levels were comparable to those observed in BLANK and Ti_6_Al_4_V derived extract incubations. Specifically, exposure to 10% of DMSO resulted in a significant decreased of cell viability compared to BLANK samples for both incubation periods. In parallel the Ti_6_Al_4_V coupon possess extremely good biocompatibility, as viability levels were minimally affected, while incubation with the Cu coupon leached media caused a dramatic decrease in L929 cell viability levels, confirming a strong cytotoxic effect and a lack of biocompatibility, due to the presence of anions leached in culture media. On the other hand, in all tested Ti–Au thin film derived leached media, cell viability levels were almost unaffected, reaching values above and/or near 80%, even after a prolonged extraction period of 168 h, suggesting a non-cytotoxic effect and a strong biocompatibility profile.

The individual elements of Ti and Au are well known and established biocompatible materials. Specifically, in the as-grown state, the amorphous nature of the Ti–Au intermetallics inhibit the ionisation of Au or Ti when immersed in the medium, thereby maintaining a good biocompatibility profile with cell viability values observed to be above 90%. Similarly, following ex-situ heat treatment, when crystallization gives rise to various intermetallics, no cytotoxic activity is observed as cell viability levels remain above 90% for all Ti–Au films. In this context, interestingly Ti rich samples S_0.10_, S_0.20_ S_0.24,_ S_0.83_ and S_1.0_ were found to further stimulate L929 cell proliferation rates, especially for 168 h extract samples, as viability levels were observed to be above 100% compared to untreated (BLANK) samples, indicating favourable conditions for cell multiplication [99,100]. In parallel, although L929 cell viability levels slightly reduced (20%) in Au rich thin films (S_0.53_ and S_0.70_), especially for 168 h extract samples, they are still considered to be biocompatible, as according to ISO 10993-5 (2009), thin film samples are characterized as cytotoxic only when they cause a decrease of cell viability below 30% [50,101].

**Fig. 14:**
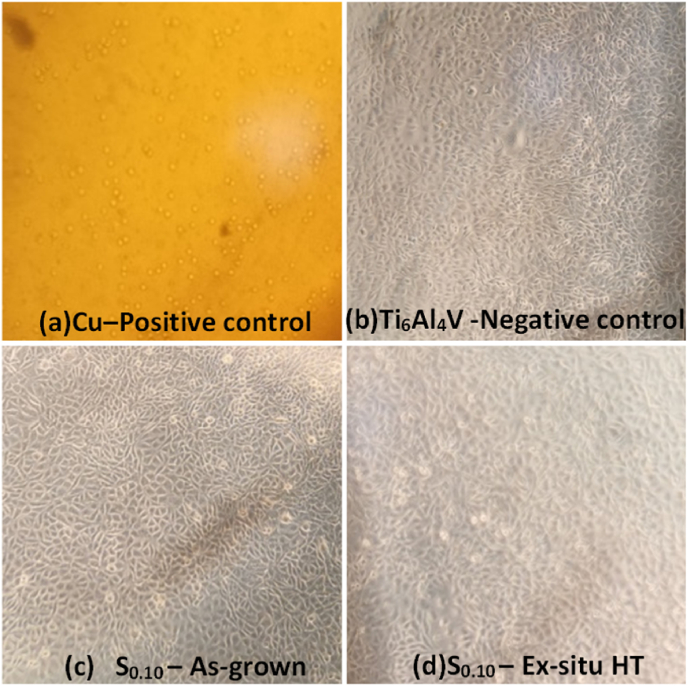
Morphological changes of L929 mouse fibroblast cells following 72 h of exposure to 168 h extracts from (a) Negative Ti_6_Al_4_V control, (b) Positive Cu control (c) Ti–Au S_0.10_ film in as-grown and (d) ex-situ heat treated states. Images acquired using an inverted Kern microscope with attached digital camera and 10X lens.

In addition to the cell viability results, alterations in L929 mouse fibroblast morphology were observed upon treatment with Cu coupon extracts (Fig. 14a), as opposed to the morphology of cells incubated with 168 h extracts from Ti_6_Al_4_V coupons (Fig. 14b) and S_0.10_ Ti–Au thin films both in their as-grown (Fig. 14c) and ex-situ HT (Fig. 14d) states. Specifically, treatment with Cu coupon extracts led to a significant reduction of cells' confluence, affecting morphology and shape, ultimately resulting in cell shrinkage and death. In contrast, Ti_6_Al_4_V coupon and S_0.10_ Ti–Au thin film extracts did not alter either the confluence or morphology of L929 cells, confirming their safe cytotoxic and biocompatible profile.

To understand the difference in cell viability results between the Ti–Au thin films and the Cu and Ti_6_Al_4_V control coupons, the concentration of ions released in each extract were tested using ICP-OEMS. The detected values of each extract were measured against calibration curves made by preparing Ti, Au, Al, V and Cu standards in 1, 5 and 10 ppm concentrations. No significant ion leaching was observed for any of the Ti–Au thin film extracts tested. The highest Au concentration of 0.024 ppm was observed for the S_0.53_ film extract, and the highest Ti concentration of 0.299 ppm was measured for the S_0.00_ film extract. Any Al or V ions observed in the extract solution are expected to originate from the underlying Ti_6_Al_4_V substrate and the highest Al concentration of 0.343 ppm was measured for the S_0.24_ film, while no V was detected during the ICP-OEMS tests. The combined concentration of Ti, Au, Al and V ions was lower than 0.728 ppm for all Ti–Au thin film extracts tested, both as-grown and following ex-situ heat treatment. However, significant leaching of Cu ions was observed from the Cu control coupon, as the anion concentration was 112 ppm. These results confirm the extremely low L929 cell viability levels (1.21%) in incubations with Cu-leached culture media, as well as previous studies indicating that an excess of 10 ppm of Cu anion concentration in leached media was found to be cytotoxic [32,33,102]. Overall, results thus far provide evidence that the addition of Au to Ti enhances the mechanical properties in the Ti rich region of Ti–Au alloys, improving the biocompatible nature of these thin film materials.

### 3.2 Ti–Au thin films grown with in-situ Tsub

#### 3.2.1 Structural and chemical characterization

It is more than evident now that oxidation plays a crucial role in affecting the mechanical properties of Ti–Au thin films when they are heat treated outside of the deposition chamber (ex-situ HT). Therefore, in order to analyse the effect that the absence of oxidation would have on mechanical and biomedical properties, five additional samples of Ti rich Ti–Au thin films (S_0.00_, S_0.10_, S_0.20_, S_0.24_ and S_0.28_) were deposited inside the nano-PVD chamber using the same deposition parameters, except that this time the substrate was heated to an elevated temperature of 450 °C using an in-situ substrate heater (in-situ Tsub). As the substrate is kept in a vacuum while raising its temperature, oxidation of the growing Ti–Au thin films can be avoided.

Table 3 summarizes the thickness and elemental composition of these five Ti–Au thin film samples. It can be seen that these in-situ Tsub films, grown at elevated substrate temperature, register higher film thicknesses compared to their as-grown counterparts after ex-situ HT (see Table 1). The Ti–Au atomic concentrations of the in-situ Tsub films are very close to the original as-grown samples and therefore their mechanical and biomedical properties can be readily compared. Elemental composition results also show absence of oxygen as required in these in-situ Tsub films.

**Table 3:** Chemical composition and thickness of Ti–Au thin films deposited with in-situ substrate temperature.

Identifier	Thickness (nm)	Elemental composition (at%)		
		Ti	Au	Ratio
**S**_**0.00**_	679	100	0	1.0 : 0
**S**_**0.10**_	718	89	11	8.1 : 1
**S**_**0.20**_	570	84	16	5.2 : 1
**S**_**0.24**_	603	75	25	3.0 : 1
**S**_**0.28**_	603	71	29	2.4 : 1

**Fig. 15:**
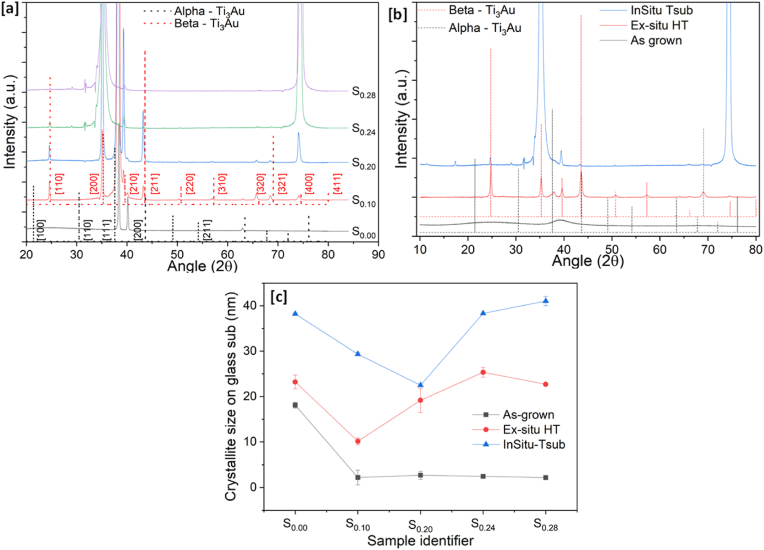
XRD patterns for (a) Ti–Au thin films grown with in-situ substrate temperature (b) comparing the quality of the β-phase of Ti_3_Au in the S_0.24_ thin film in as-grown vs ex-situ heat treated vs in-situ substrate temperature states and (c) calculated crystallite sizes for these three states.

The XRD patterns from the five in-situ Tsub samples are shown in Fig. 15 (a). All films show extremely good crystalline nature, unlike the as-grown thin films previously grown at room temperature (Fig. 4). The pure Ti thin film (S_0.00_) is observed to be in the α-phase with peak positions at 38.4°, 40.2° and 63.1°. However, with the addition of Au, S_0.10_ film is found to consist of a mixture of α and β-phases of Ti_3_Au, with a peak position of 37.9° located at a slightly higher angle than the expected location of the (111) orientation of the α-phase. The S_0.10_ film contains 11 at% of Au, which is less than the 3:1 ratio required to form phases of Ti_3_Au, but the energy provided by the substrate temperature enables the unit cell arrangement of the crystalline Ti_3_Au intermetallic structure to form. Therefore, the resulting α-phase of Ti_3_Au, is under stress, as the smaller Ti atoms (Radii = 0.081 nm) start to replace the sites which should have been occupied by the larger Au atoms (Radii = 0.099 nm), shifting the peak positions of the α-phase to higher angles [103]. The rest of the peak patterns from these samples align with that of the β-phase, with peak positions shifted to lower angles. Still, a very good match is achieved for (110), (200) and (211) at 24.6°, 35.2° and 39.5° respectively. This situation improves with further addition of Au in the system (S_0.20_), as the peak position matches very well with the β-Ti_3_Au phase. For sample S_0.24_, major peaks are positioned at 24.7°, 35.3°, 39.6° and 74.5° representing (110), (200), (210) and (400) orientations of the β-Ti_3_Au phase. This condition is based on the fact that the composition ratio of S_0.24_ is closest to Ti_3_Au and the energy provided by the substrate temperature arranges the unit cells in these preferred orientations. Finally, the S_0.28_ film also exhibits a crystal structure very similar to that of the S_0.24_ film. The diffraction patterns for thin films deposited on Ti_6_Al_4_V substrates and their calculated crystallite size is presented in the supplementary section (data 5) and is in good agreement with the patterns and values from glass substrates. The improvement in structure perfection for Ti–Au thin films with in-situ heat treatment is yet again supported by the 8 to 20 times reduction in FWHM, which is better than the 5 to 10 times reduction achieved by ex-situ heat treatment.

For a better comparison of the quality of the crystalline structure formed, the diffraction pattern for the S_0.24_ thin film grown with in-situ Tsub is compared with its ex-situ HT counterpart, as provided in Fig. 15 (b). It is very clear that the broad curve representing the quasi-crystalline nature of the as-grown film turns into a multi peaked pattern indicating development of the β-phase after ex-situ HT at 450 °C. However, the diffraction pattern for the in-situ Tsub has fewer, very high intensity peaks, indicating preferential development of β-Ti_3_Au crystals in the [100] direction. The structural perfection for S_0.24_ improves considerably when compared in its as-grown, ex-situ heat treated and in-situ Tsub states, with a more than 20 times reduction in its FWHM, from 4.31° to 0.42° to 0.21°, respectively. In addition, the quality of crystal formation is also depicted in the calculated crystallite sizes for films grown with in-situ Tsub compared against as-grown and ex-situ HT samples, as shown in Fig. 15 (c). As discussed earlier, the as-grown pure titanium (S_0.00_) sample has a larger crystallite size of 18 nm, which is further reduced to values lower than 3 nm when Au is introduced in the film (S_0.10,_ S_0.20,_ S_0.24_ and S_0.28_). Following ex-situ HT, the pure Ti thin film shows a slight increase in crystallite size to 23 nm, whereas the rest of films register higher increases in size due to development of the Ti_3_Au crystalline phase. However, it is evident that the calculated crystallite sizes for the thin films grown with in-situ Tsub are significantly larger (Fig. 15c). Even though there is a reduction in the crystallite size from S_0.00_ to S_0.10_ and S_0.20_ before it starts to increase again from S_0.24_ onwards, all the values of crystallite size for in-situ Tsub samples are larger than their ex-situ HT counterparts. This improvement in crystallite size is a result of higher intensity diffraction peaks arising from improved crystallization of the Ti_3_Au intermetallic at elevated substrate temperature, without the negative effects of oxidation seen in the ex-situ heat treated samples.

#### 3.2.2 Morphology characterization

**Fig. 16:**
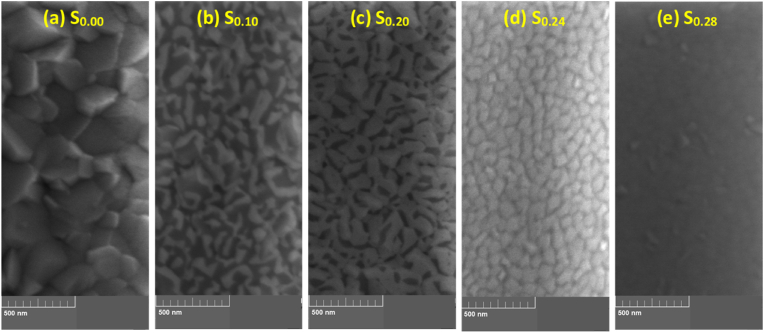
Surface SEM images of Ti–Au thin films deposited with in-situ substrate temperature on glass substrates with increasing Au composition.

The surface topology of Ti–Au thin films grown with in-situ Tsub of 450 °C is shown in Fig. 16 (a to e). Specifically, a clear distinction can be seen in these images when compared to the surface morphology observed in the as-grown (Fig. 5) or ex-situ HT (Fig. 7) samples. The pure Ti thin film (S_0.00_) deposited with in-situ Tsub retains the polyhedral shape but the grains are larger with no voids in between the boundaries. However, sample S_0.10_ develops a light and a dark region which can be very clearly distinguished in the surface images, while with a further increment in Au for S_0.20_, the lighter region begins to expand and gains almost full coverage for the S_0.24_ sample. S_0.28_ sample reveals a film morphology with an extremely dense glass like appearance, with randomly dispersed small surface features. EDX analysis revealed no distinction between the elemental composition of the dark and the light phase, however, correlating with XRD patterns (Fig. 15), the darker region can be assigned to the α-phase of the Ti_3_Au intermetallic alloy, while the light region to its β-phase. With the increment of Au from 10 to 24 at% in the film, the conditions become more suitable for growth of the β-phase. The quality of α and β-phase achieved by in-situ Tsub (Fig. 16) is much better than those observed earlier with ex-situ HT samples (Fig. 7).

**Fig. 17:**
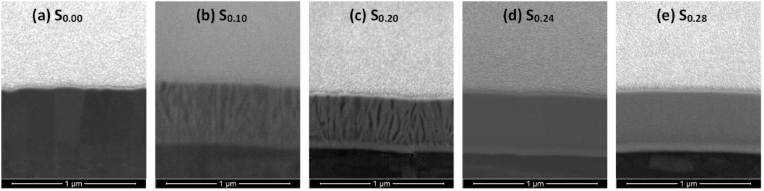
Focussed ion beam (FIB) cross section images of Ti–Au thin films (a) S_0.00_ (b) S_0.10_, (c) S_0.20_, (d) S_0.24_ and (e) S_0.28_ deposited on Ti_6_Al_4_V substrates at elevated In-situ Tsub.

The high quality of the Ti–Au intermetallic formed when deposited with in-situ Tsub is also reflected in the cross-section FIB images of thin films S_0.00_, S_0.10_, S_0.20_, S_0.24_ and S_0.28_ shown in Fig. 17 (a to e). The columns of Ti extending throughout the film thickness are again visible for the pure Ti film (S_0.00_) grown with in-situ Tsub (Fig. 17a). For ex-situ HT sample S_0.10_, the columnar structure of the Ti–Au intermetallics only developed partially across the film thickness, with oxide layer contamination on the surface when the thin film was heat treated externally in a tube furnace (Fig. 8b). However, when the same thin film sample was deposited at elevated substrate temperature, the columnar structure of α and β phases (dark and light regions) develops across the full film thickness without any surface oxide formation (Fig. 17b). With further addition of Au in the film, in sample S_0.20_ (Fig. 17c), the lighter columns representing the β phase become dominant over the darker α regions. No such columnar structure exists for samples S_0.24,_ and S_0.28_ which maintain their densely packed fined grained structure with preferential development of the light coloured β-Ti_3_Au phase as the desired 3:1 stoichiometric ratio is achieved (Fig. 17d and e). When correlated with the XRD results (Fig. 15) and surface morphology (Fig. 16), these cross-sectional images support the claim that a better crystal structure of the β-Ti_3_Au intermetallic compound is developed when the Ti–Au thin films are grown at elevate substrate temperature instead of external heat treatment in a tube furnace.

**Fig. 18:**
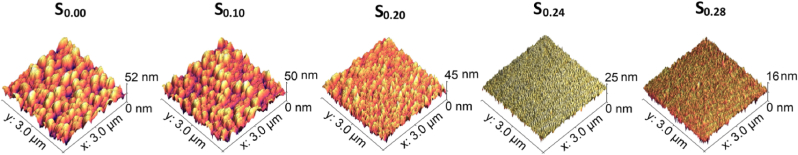
AFM micrographs of Ti–Au thin films deposited with in-situ substrate temperature on glass substrates with increasing Au composition.

AFM micrographs of thin films grown with in-situ Tsub are shown in Fig. 18, while their corresponding measured surface roughness values are summarized in Table 2. Owing to better crystallization dynamics and lower void formation, the pure Ti thin film deposited with in-situ Tsub registers smaller oval shaped feature sizes in the range of 52 nm, when compared to the as-grown film seen earlier, which has larger triangular shaped grains of around 76 nm. Comparatively, the Ti rich samples such as S_0.10_ and S_0.20_ exhibit sharper peaks and smaller feature sizes extending up to 50 and 45 nm respectively. With further increase in Au, samples S_0.24_ and S_0.28_ exhibit needle like structures with feature sizes of only 25 and 16 nm, respectively. Surface roughness is also seen to gradually decrease from 7 nm to less than 1 nm with increase in Au concentration in these films grown with in-situ Tsub (Table 2).

#### 3.2.3 Mechanical characterization

**Fig. 19:**
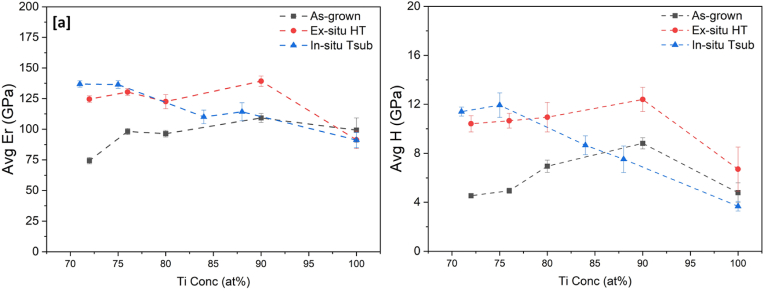
Variation of (a) Reduced elastic modulus (Er) and (b) Hardness (H) of Ti–Au thin films with increasing Au composition, deposited on glass substrates with in-situ substrate temperature compared with results from as-grown and ex-situ heat treated samples.

Reduced elastic modulus and hardness of the Ti–Au thin films deposited with in-situ Tsub on glass substrates is compared with their as-grown and ex-situ HT counterparts in Fig. 19 (a) and (b), respectively. The pure Ti films (S_0.00_) grown with in-situ Tsub register a reduced elastic modulus value of 91 ± 6 GPa, which increases steadily with each increment in Au concentration, reaching a maximum value of 136 ± 2 GPa for S_0.28_ sample. The hardness of these films also increases steadily from 3.6 ± 0.3 GPa for pure Ti thin films to a peak value of 11.9 ± 0.9 GPa for S_0.24_ and shows a decreasing trend thereafter. The hardness peak achieved at 3:1 atomic ratio of Ti:Au is more repeatable and reliable, as the uncontrolled surface oxidation seen for the ex-situ HT samples is avoided, and instead better crystallization of β-Ti_3_Au is achieved by controlling the substrate temperature during film growth. The hardness of the Ti_3_Au intermetallic system is greatly affected by the ratio of its α and β-phases at any given time, as this will affect the length of the grain boundaries. As the atomic bonds become strengthened along these grain boundaries, the resistance to dislocation propagation will also improve, thereby increasing the materials hardness [28,80]. Such increase in grain boundaries due to reduction of α towards β-phase can be clearly visualised in the SEM images shown in Fig. 16. Mechanical results for the TiAu thin films grown on Ti _6_Al_4_V substrates are very similar to the results on glass substrates and are provided in the supplementary section (data 6). Moreover, in the case of films on Ti_6_Al_4_V substrates, the highest reduced elastic modulus value of 164 ± 15 GPa is observed for sample S_0.28_ and the peak hardness value of 11.5 ± 2 GPa for sample S_0.24_. Increase in elastic modulus due to substrate effects and increased deviation of the achieved results due to larger surface roughness is once again clearly observed for films deposited on Ti_6_Al_4_V substrates.

#### 3.2.4 Cytotoxicity-biocompatibility tests on L929 cells

Cytotoxicity results on L929 mouse fibroblast cells following exposure to extracts from Ti–Au thin films grown with in-situ Tsub are summarized in Table 4. As expected, an extreme cytotoxic effect was observed for Cu coupon and 10% DMSO treatments, as opposed to the unaffected viability levels in the case of BLANK and Ti_6_Al_4_V coupon incubations. Similar to the safe cytotoxic profile of Ti–Au thin films in their as-grown ex-situ HT states (Fig. 13), extracts from Ti–Au thin films grown with in-situ Tsub, obtained after both 72 h and 168 h, produced an excellent biocompatible profile, as viability levels of L929 cells were minimally affected following 72 h of exposure, reaching values between 80 and 100% for all tested samples (Fig. 14). The leached Ti, Au, Al, V and Cu ion concentrations from these in-situ Tsub films were also better than 0.5 ppm, again matching very closely to the as-grown and ex-situ HT samples. Morphological changes of L929 cells following 72 h incubation, captured using an inverted Kern microscope, support the biocompatible profile of Ti–Au thin films deposited with in-situ substrate temperature. Comparative images between the controls and one of the Ti–Au thin film compositions are presented in the supplementary section (data 7).

**Table 4:** Cell viability levels of L929 mouse fibroblast cells following 72 h of exposure to 72 h and 168 h extracts from Ti–Au thin films deposited with in-situ substrate temperature of 450 °C, compared to negative controls (untreated (BLANK) and Ti_6_Al_4_V coupon) and positive controls (Cu coupon and 10% DMSO).

Sample identifier		72 h		168 h	
		Viability Mean (100%)	Std. Dev	Viability Mean (100%)	Std. Dev
Controls	Blank DMEM	96.7	1.0	95.8	1.6
	10% DMSO	<0	1.8	<0	1.7
	Cu coupon	1.0	1.0	1.0	1.0
	Ti_6_Al_4_V coupon	102.9	8.8	104.8	8.7
Ti–Au thin films	S_0.00_	88.0	7.0	79.1	5.3
	S_0.10_	87.2	2.5	86.4	3.9
	S_0.20_	92.2	1.2	80.5	5.7
	S_0.24_	102.4	9.5	91.5	2.4
	S_0.28_	87.7	2.1	89.5	3.0

## 4 Conclusion

This work has successfully demonstrated the means to enhance the mechanical properties of Ti–Au alloy thin film materials to achieve hardness values better than 12 GPa, while retaining the excellent biomedical properties from the individual Ti and Au elements. Ti–Au thin films deposited on glass or Ti_6_Al_4_V substrates at room temperature are quasi-crystalline in nature with broad diffraction patterns covering the full spectrum of Ti–Au intermetallic peak positions. With addition of Au, the polyhedral shape of the as-grown pure Ti film gives rise to an extremely fine textured Ti–Au intermetallic surface with a corresponding low surface roughness. In the case of as-grown films, the reduced elastic modulus reaches a peak value of 139 GPa, together with a moderate hardness of 8.8 GPa for samples with very low Au concentration of 10 at%. On the other hand, ex-situ heat treatment at 450 °C leads to improved crystallization of the Ti–Au intermetallics with α and β-T_i3_Au developing for Ti rich regions (10 < Au at% > 34). Heat treatment also leads to the formation of amorphous titanium oxides in the upper surface with migration of Ti from underlying regions. A maximum hardness of 12.3 GPa is achieved for TiAu films containing 10 at% Au after ex-situ heat treatment. This Au concentration of 10 at% is much lower than that of 25 at% observed in previous work to achieve similar hardness results and correlates to a combined effect of surface oxidation, defect healing, phase crystallization and grain growth. Similar mechanical performance at lower Au concentration would also have economic benefits for the application of Ti–Au thin film coatings for long lasting biomedical implants. In comparison, thin films deposited with in-situ elevated substrate temperature of 450 °C show a superior quality of crystallization of α and β-phases of the Ti_3_Au intermetallic, while also achieving a similar peak mechanical hardness of 11.9 GPa. Moreover, In-situ heat treatment is a much simpler single stage production process to produce high quality Ti–Au films and avoid surface oxidation, which can be an uncontrolled source of defect. Finally, all types of Ti–Au thin films deposited in this work are found to be highly biocompatible show a non-cytotoxic effect on L929 mouse fibroblast cells. Specifically, viability levels following incubations with all Ti–Au films were found to be between 80 and 100% compared to BLANK (untreated cells), even after a prolonged extraction time of 168 h, while a number of samples were capable of further stimulating L929 cell proliferation rates. By gaining control over crystallization quality and introduction of interstitial species, we have demonstrated a simple single stage scalable method to produce Ti_3_Au thin films with enhanced mechanical and biological performance. With further research and development, this novel material has the potential to be the next generation super-hard, biocompatible coating to extend the lifetime of orthopaedic implants.

## CRediT authorship contribution statement

**Cecil Cherian Lukose:** Investigation, Formal analysis, Validation, Data curation, Visualization, Writing – original draft. **Ioannis Anestopoulos:** Investigation, Formal analysis, Validation, Writing – review & editing. **Theodora Mantso:** Investigation, Formal analysis, Validation. **Leon Bowen:** Methodology, Investigation, Formal analysis. **Mihalis I. Panayiotidis:** Conceptualization, Methodology, Writing – review & editing, Supervision, Funding acquisition. **Martin Birkett:** Conceptualization, Methodology, Writing – review & editing, Supervision, Project administration, Funding acquisition.

## Declaration of interests

The authors declare that they have no known competing financial interests or personal relationships that could have appeared to influence the work reported in this paper.
