# Supplementary material for: Thermal activation of Ti(1-x)Au(x) thin films with enhanced hardness and biocompatibility
Source: Bioact Mater. 2022 Mar 3;15:426–45. doi: 10.1016/j.bioactmat.2022.02.027 (PMC8958427; doi:10.1016/j.bioactmat.2022.02.027)

**Supplementary Data**

1. Supplementary data 1: EDX spectra’s of (a) 10 As-grown samples (b) Top layers of 10 Ex-situ heat treated samples (c) Bottom layers of 10 Ex-Situ heat treated samples (d) 6 In-situ heat treated samples.


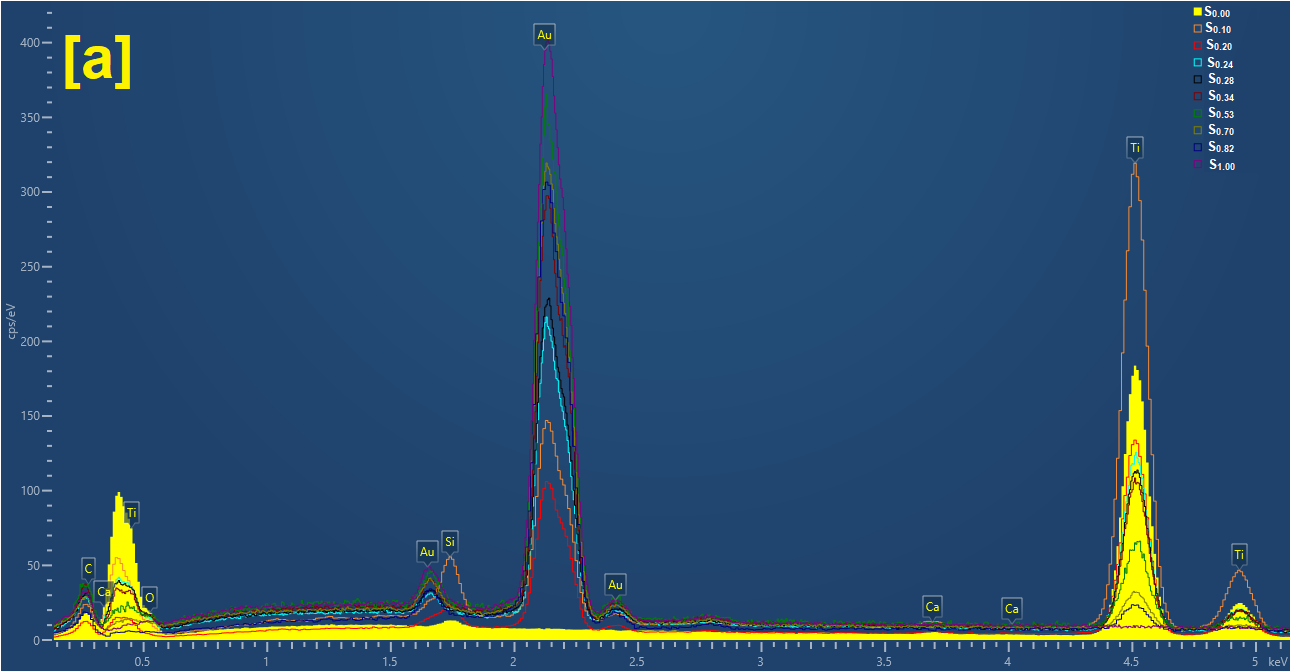


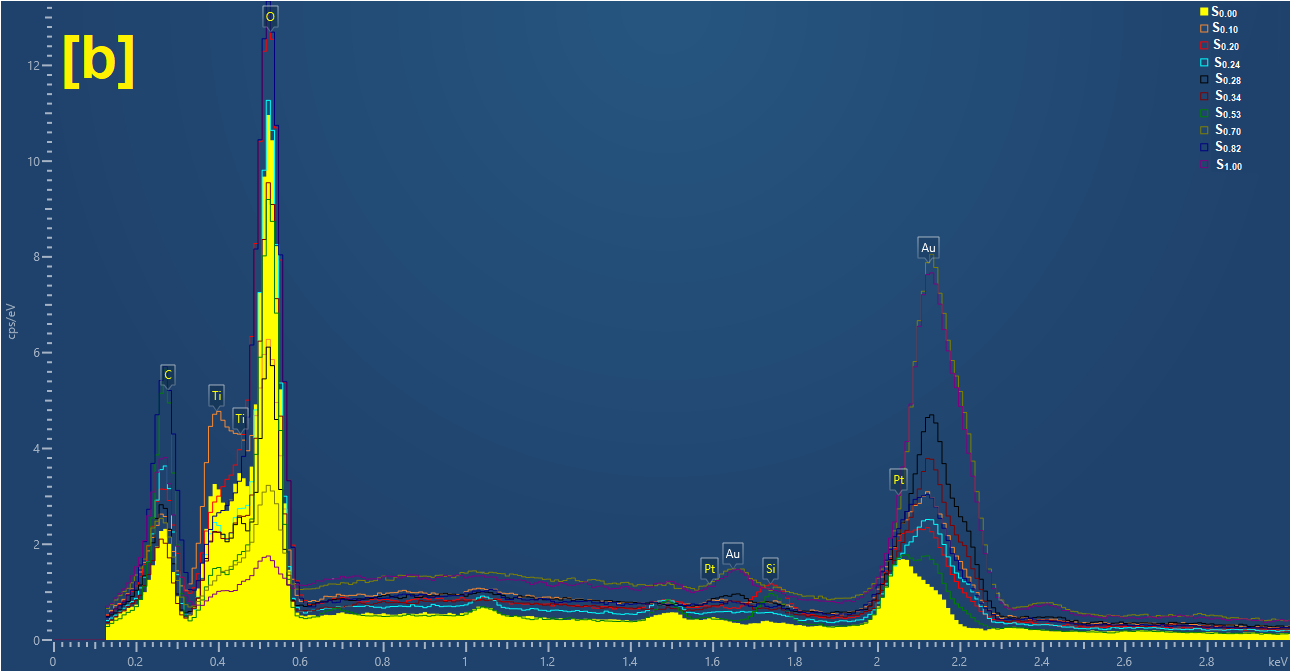


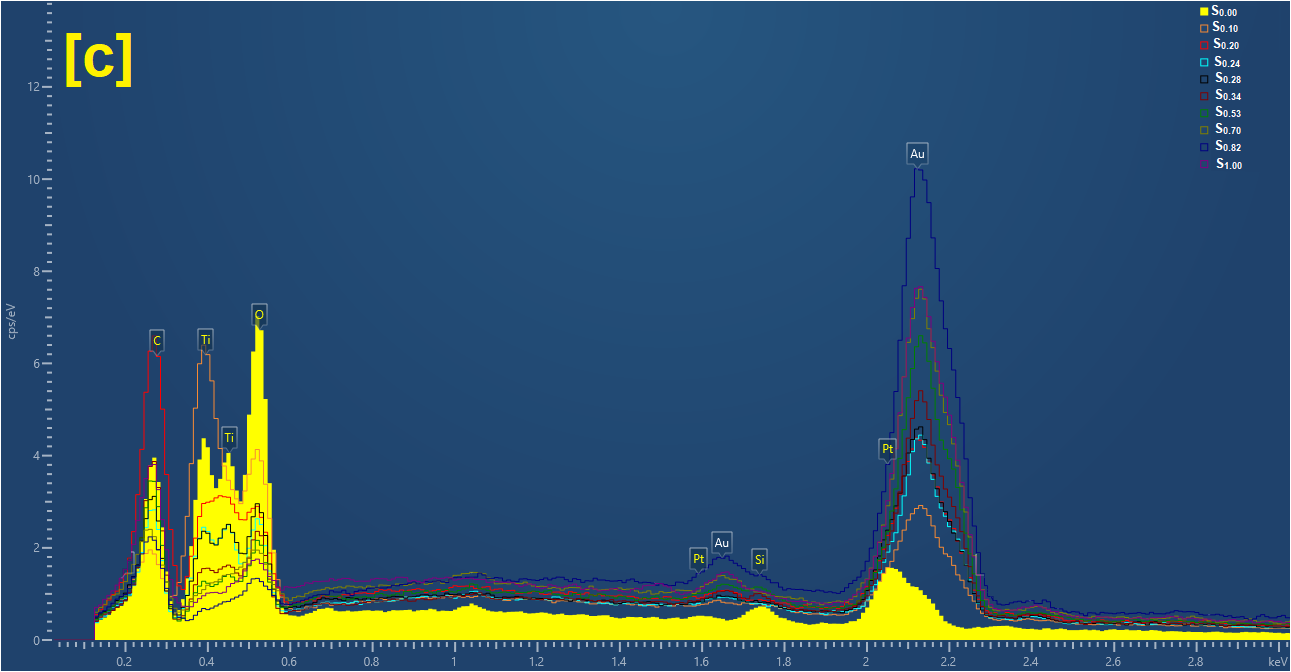


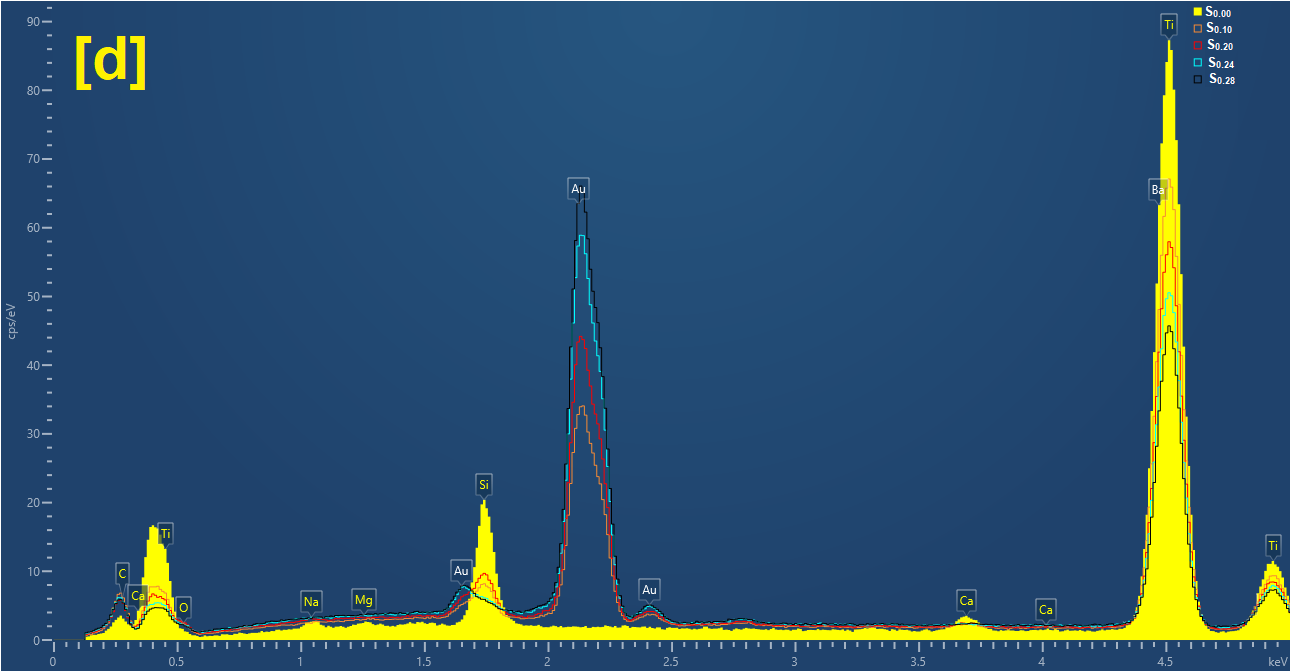


1. Supplementary Data 2: XRD patterns for (a) as-grown and (b) Ex-situ heat treated Ti-Au thin films and (c) crystallite size calculated for these films deposited on Ti substrates.


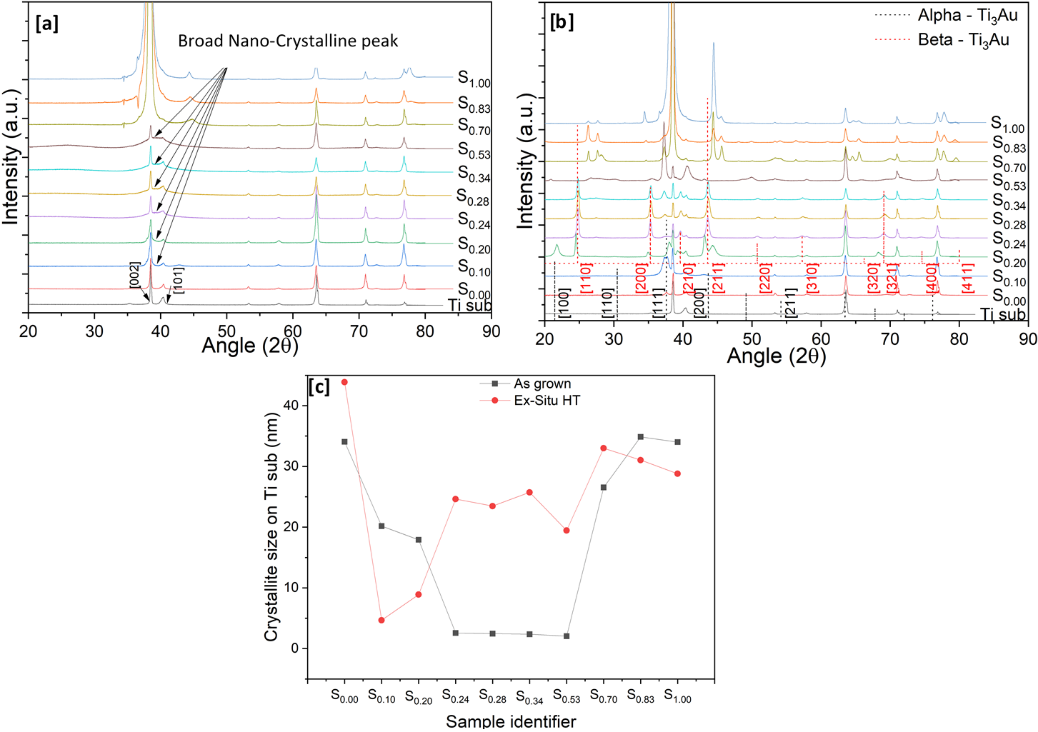


1. Supplementary data 3: (a) Micro-strain calculated for as-grown, ex-situ heat treated and in-situ Tsub samples using Scherrer’s method, shows the relaxation achieved in Ti rich films after heat treatment. Due to their quasi-crystalline nature, as-grown samples were not suitable for the W-H plot method and had to be performed using Scherrer’s method and so results calculated from ex-situ and in-situ using the same method were included for comparison. (b) Micro strain calculated for ex-situ samples (black line) using the W-H plot method because of their improved crystallinity, shows that values remain comparable to those measured with Scherrer’s method. And even with this method, In-situ heat Tsub samples (red line) achieve better stress relaxation when compared to ex-situ samples.


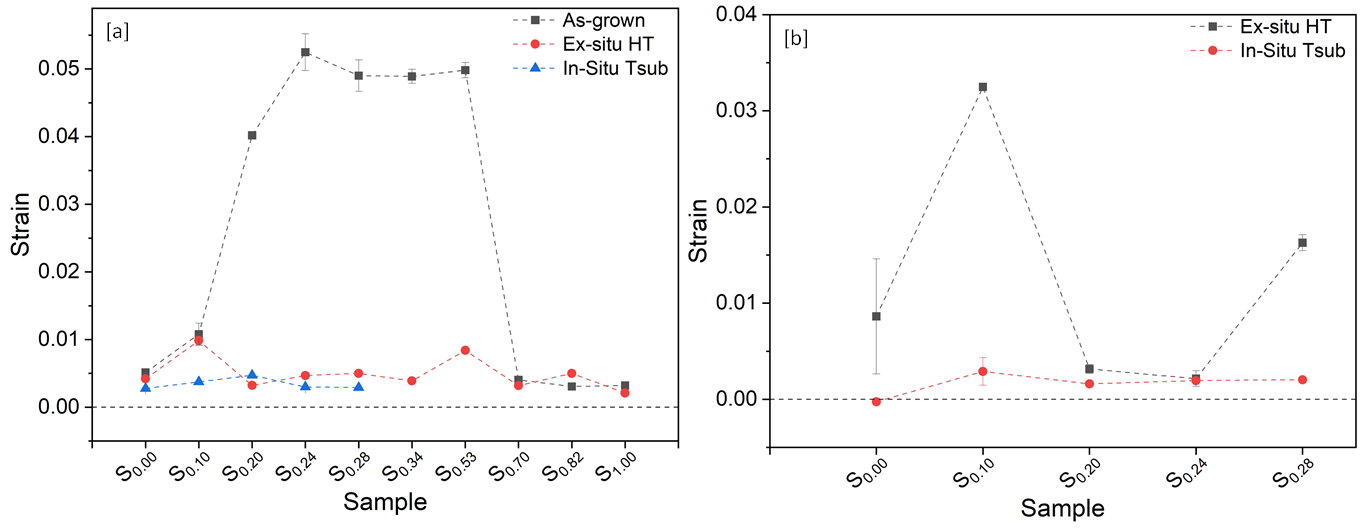


1. Supplementary data 4: Cross sectional SEM images of (a) as-grown (b) Ex-situ heat treated and (c) in-situ Tsub Ti-Au thin films


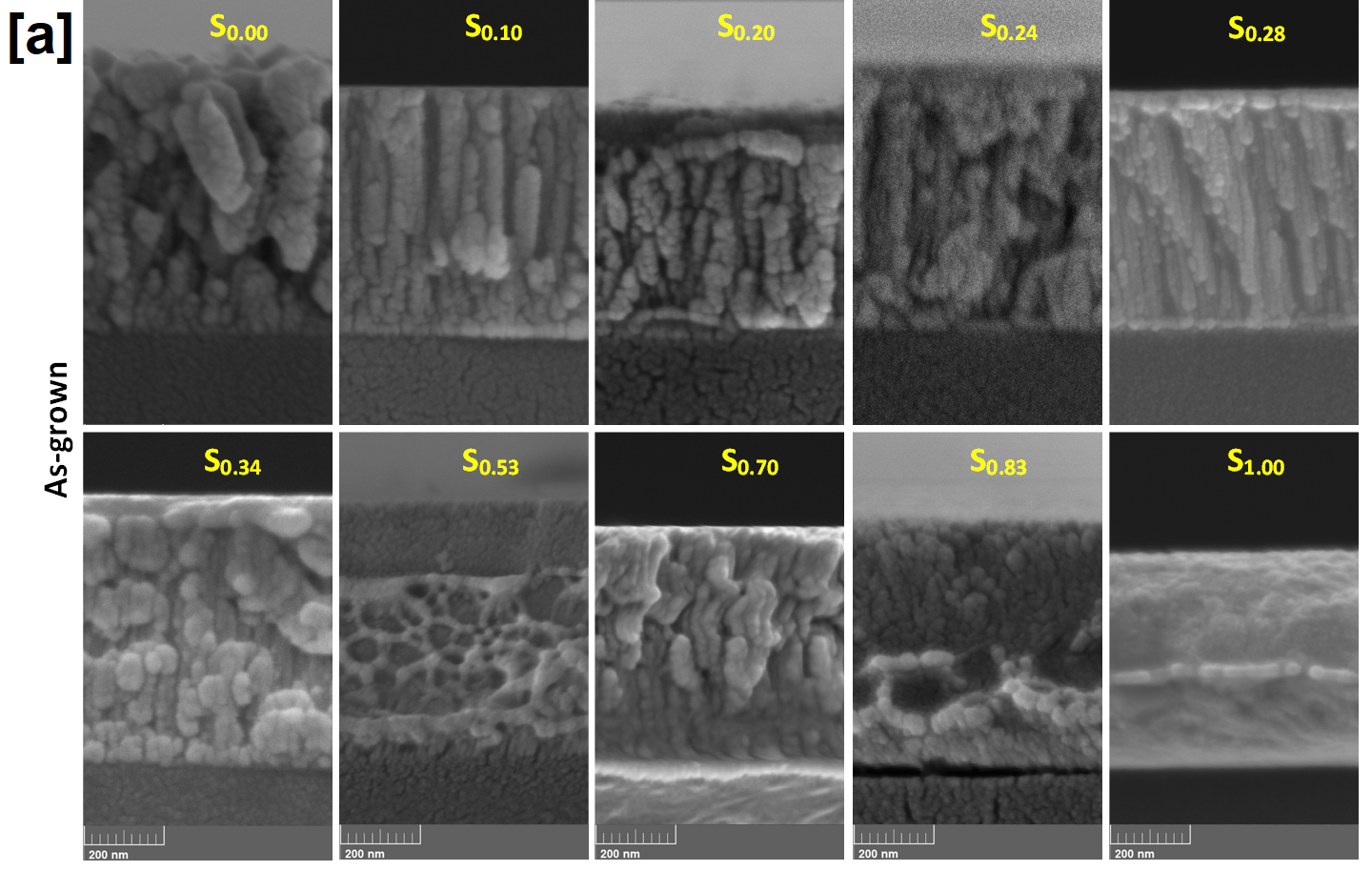


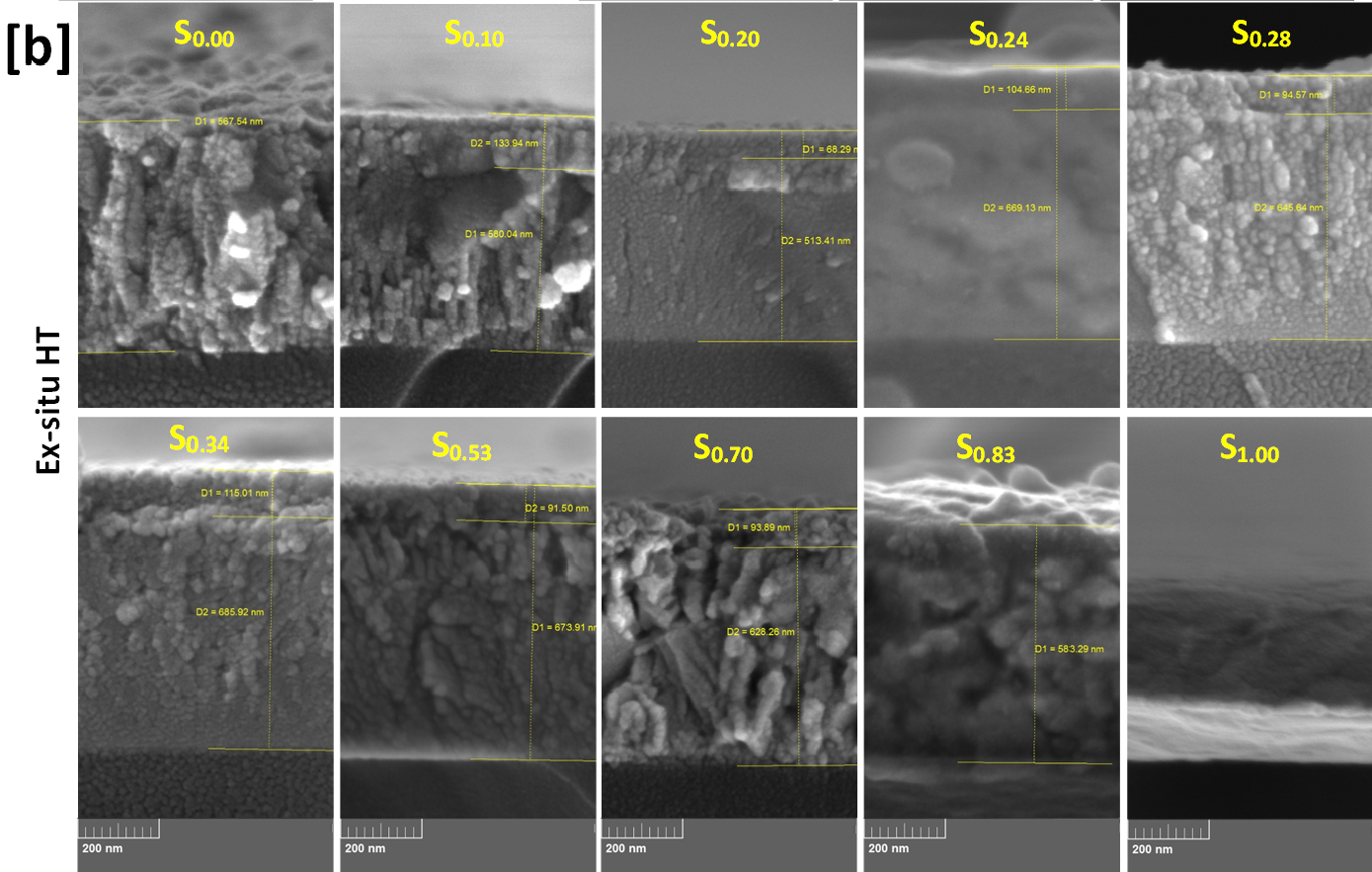


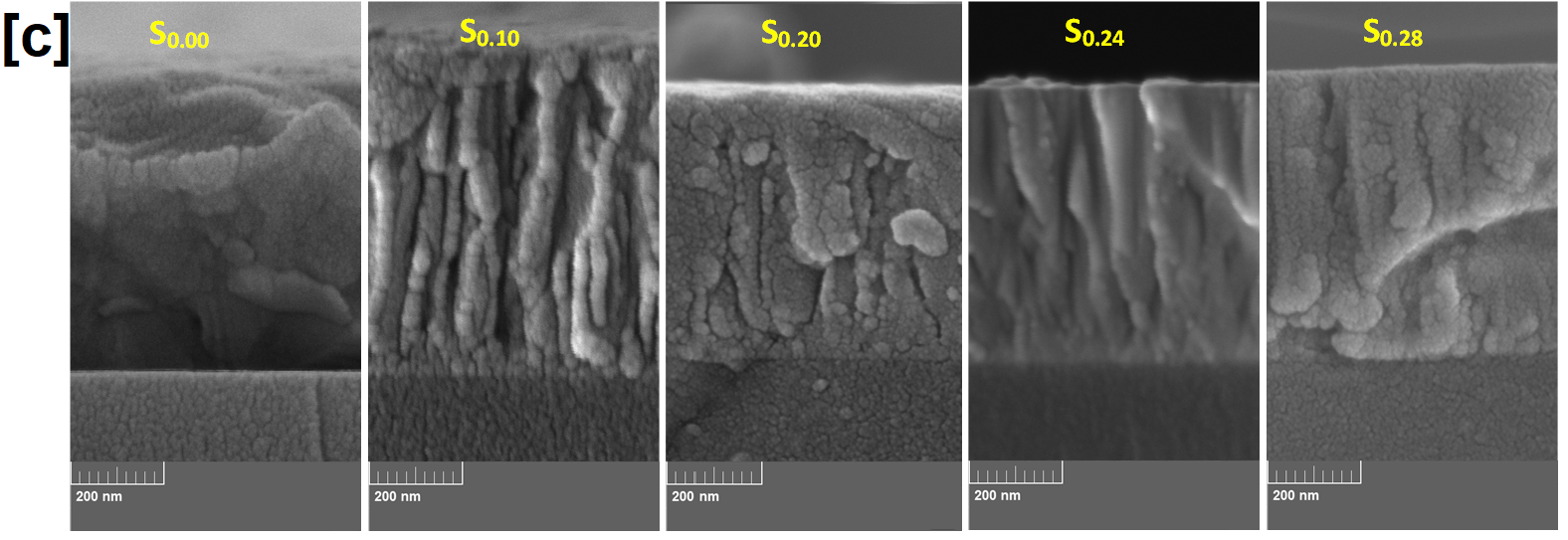


1. Supplementary Data 5 : XRD patterns for (a) Ti-Au thin films grown with elevated in-situ substrate temperature, (b) comparing as-grown vs ex-situ heat treated vs in-situ heat treated S_0.24_ thin film, showing the quality of the β-phase of Ti_3_Au grown in each case and (c) calculated crystallite sizes, on Ti substrate.


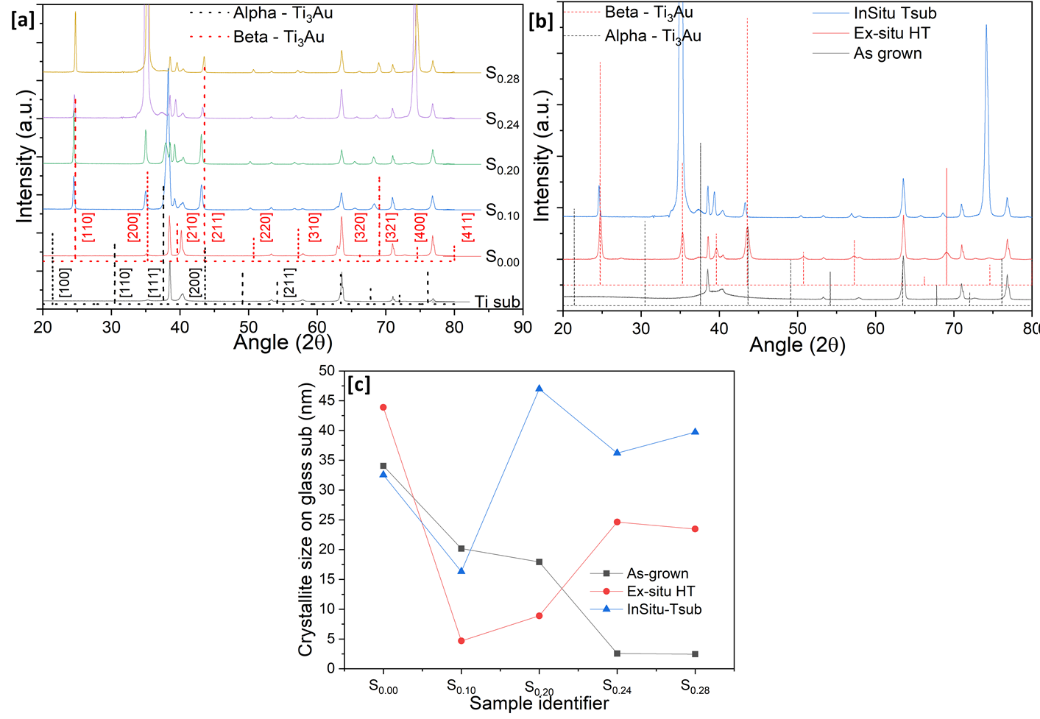


1. Supplementary Data 6: Variation of (a) Elastic modulus (Er) and (b) Hardness of Ti-Au thin films deposited on Ti substrates at elevated in-situ substrate temperature, compared with the results obtained from as-grown and ex-situ heat treated samples.


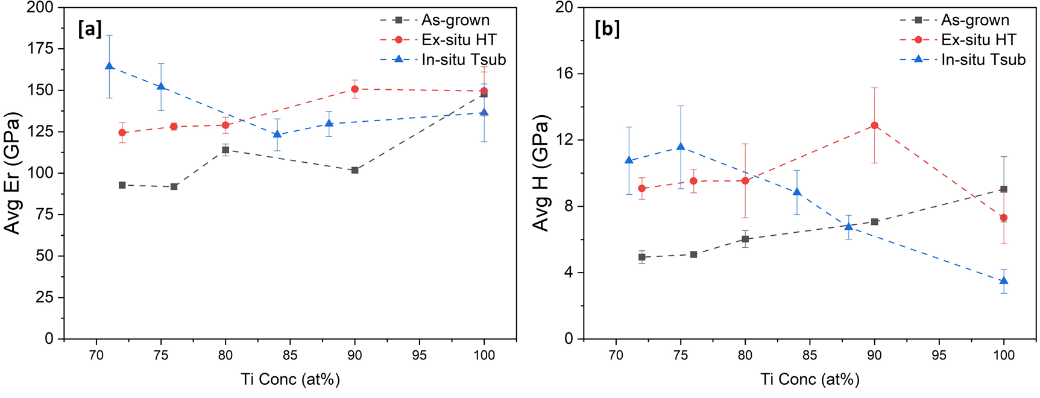


1. Supplementary Data 7: Morphological changes of L929 mouse fibroblast cells following 168h incubations with extracts from (a) In-situ Tsub S_0.10_ thin film sample, (b) Positive Cu control (c) as-grown S_0.10_ thin film sample and (d) ex-situ heat treated S_0.10_ thin film sample. Images were acquired using an inverted Kern microscope with attached digital camera and 10X lens.


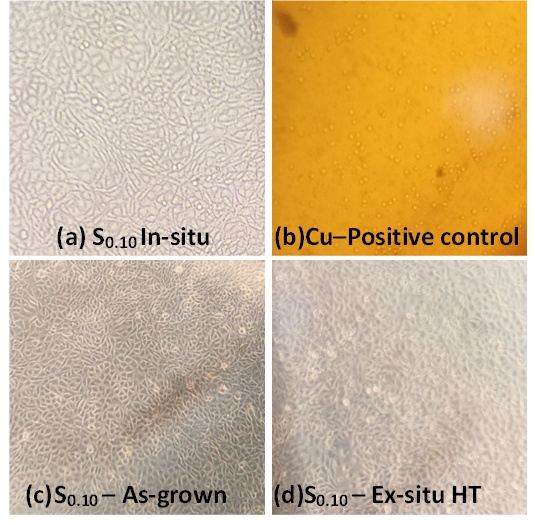

Supplement: Multimedia component 1 [file mmc1.docx]
